# Effects of Menu Labeling Policies on Transnational Restaurant Chains to Promote a Healthy Diet: A Scoping Review to Inform Policy and Research

**DOI:** 10.3390/nu12061544

**Published:** 2020-05-26

**Authors:** Sofía Rincón-Gallardo Patiño, Mi Zhou, Fabio Da Silva Gomes, Robin Lemaire, Valisa Hedrick, Elena Serrano, Vivica I. Kraak

**Affiliations:** 1 Department of Human Nutrition, Foods, and Exercise, College of Agriculture and Life Sciences, Virginia Polytechnic Institute and State University, Blacksburg, VA 24061, USA; mi14@vt.edu (M.Z.); vhedrick@vt.edu (V.H.); serrano@vt.edu (E.S.); vivica51@vt.edu (V.I.K.); 2 Department of Non-Communicable Diseases and Mental Health, Pan American Health Organization, World Health Organization, Washington, DC 20037, USA; gomesfabio@paho.org; 3 Center for Public Administration and Policy, School of Public and International Affairs, Virginia Polytechnic Institute and State University, Blacksburg, VA 24061, USA; rlemaire@vt.edu

**Keywords:** food labeling, menu labeling, nutrition declaration, food and nutrition policy, restaurant chains, reformulation, serving size, energy, obesity

## Abstract

There is insufficient evidence that restaurant menu labeling policies are cost-effective strategies to reduce obesity and diet-related non-communicable diseases (NCDs). Evidence suggests that menu labeling has a modest effect on calories purchased and consumed. No review has been published on the effect of menu labeling policies on transnational restaurant chains globally. This study conducted a two-step scoping review to map and describe the effect of restaurant menu labeling policies on menu reformulation. First, we identified national, state, and municipal menu labeling policies in countries from global databases. Second, we searched four databases (i.e., PubMed, CINHAL/EBSCO, Web of Science, and Google Scholar) for peer-reviewed studies and gray-literature sources in English and Spanish (2000–2020). Step 1 identified three voluntary and eight mandatory menu labeling policies primarily for energy disclosures for 11 upper-middle and high-income countries, but none for low- or middle-income countries. Step 2 identified 15 of 577 studies that met the inclusion criteria. The analysis showed reductions in energy for newly introduced menu items only in the United States. We suggest actions for governments, civil society organizations, and the restaurant businesses to develop, implement, and evaluate comprehensive menu labeling policies to determine whether these may reduce obesity and NCD risks worldwide.

## 1. Introduction

Unhealthy dietary patterns characterized by the rapid nutrition transition are associated with obesity and diet-related non-communicable diseases (NCDs) [1]. Over the past several decades, dietary patterns have shifted from eating home-cooked meals to eating out more frequently [2,3]. Eating away from home is linked to an increased consumption of ultra-processed food and beverage products with excessive calories, fat, and added sugars and sodium [4,5,6,7]. Cafeterias, fast-food restaurant chains, independent take-out-restaurants, and food retailers contribute substantially to the daily energy intake [8,9]. A global survey conducted with 30,000 online respondents across 61 countries found that 48% of participants reported eating away from home weekly or more often with quick-service restaurants (QSRs) and fast-casual restaurants (FCRs) being the most preferred [10]. 

Evidence suggests that food labeling at point-of-purchase may inform shoppers to choose healthier options [11,12,13]. The World Health Organization (WHO) has recommended nutrition labeling and reducing portion sizes as strategies to reduce energy intake; however, there is insufficient evidence to show that menu labeling legislation for chain restaurants and food retailers is a cost-effective “best buy” policy to improve diet quality and reduce NCD-related disability and mortality in low- and middle-income countries [14].

The aim of menu labeling policies is to reduce energy intake and improve diet quality by helping consumers make better-informed decisions and to encourage food retailers and restaurant businesses to reformulate menu items and reduce and standardize serving sizes to meet recommended nutrient targets [15,16]. This dual goal has the potential to improve the nutrition and diet quality of individuals who eat away from home frequently because it may impact entire populations and does not require conscious individual behavior changes [17,18]. 

The restaurant business sector, which includes QSRs, FCRs, and full-service restaurant (FSR) chains and independent restaurants, has the resources and capacity to reformulate menu items or introduce new items [19,20,21]. United States (US) chain restaurant establishments have demonstrated progress to improve the nutrition composition of items and reduce meal size or portions served to meet recommended nutrient targets of public health experts, namely, the United States Department of Agriculture and the Dietary Guidelines for Americans [20]. A systematic review conducted in 2019 identified trends for restaurant chains to reformulate food and beverage products and reduce or standardize portions in 30 countries across six regions worldwide between 2000 and 2018 [21]. Recommendations by public health practitioners have been issued to downsize and standardize portions to 600–700 calories or 2510–2930 kilojoules/meal as an important strategy for restaurants to help costumers reduce obesity and NCD risks. However, this research found a lack of clear, universal, and internationally accepted standards for transnational restaurant chains to adopt portion or serving sizes for meals, beverages, side dishes, and desserts served to children, adolescents and adults [21]. The studies reviewed (*n* = 50) also revealed wide variation within and across countries, regions, firms, and restaurant chains to reduce energy, saturated fats, trans fats, sodium, and standardized portions. In addition, menu labeling may influence some of the documented progress [21].

The implementation of menu labeling policies in countries has led to 12 published systematic reviews and/or meta-analyses that examined the influence of restaurant menu labeling on consumer dietary behaviors between 2008 and 2018. These studies documented a modest yet statistical reduction in calories purchased and/or consumed at chain restaurants and other food-service settings [15,22,23,24,25,26,27,28,29,30,31,32]. However, only one published literature review examined the restaurant industry’s reformulation of menu items [15]. No review has been published on whether menu labeling policies have an effect on reformulation, introduction of new or existing products, or reduction of serving sizes on menus from transnational restaurant chains globally. 

Given the lack of published evidence on this topic, a better understanding is needed of the effects of mandatory and voluntary menu labeling on the restaurant sector’s businesses. The results may be used to inform governments, civil society organizations, researchers, and the restaurant sector across countries on whether and how to develop comprehensive and robust policies that encourage industry changes to promote healthy dietary choices that will help to reduce obesity and NCD risks worldwide.

### Study Purpose

The purpose of this study is two-fold: (1) to conduct a scoping review to map and describe the menu labeling policies enacted across countries and regions from 2000 to 2020; and (2) and to examine evaluations for any measurable effects (i.e., positive, no, or mixed) that restaurant menu labeling policies have on businesses to reformulate products or introduce new products and reduce the serving size of menus items served and sold to customers. The results are discussed within the context of government actions needed to strengthen policies and invest in external monitoring and evaluations of menu labeling legislation. We also discuss the need to make a compelling business case to encourage restaurant businesses to reformulate menu items to meet recommended healthy nutrient targets. This objective is part of a broader marketing-mix choice-architecture approach to improve their corporate image and attract new customers interested in health and wellness. Finally, we examine the implications for actions for diverse stakeholders, including governments, the WHO, restaurant businesses, private foundations, researchers, and civil society organizations to develop comprehensive menu labeling policies to determine whether these may reduce obesity and NCD risks worldwide.

## 2. Materials and Methods

This study was a two-step scoping review, conducted between 1 January and 29 February 2020 to examine the influence of restaurant menu labeling policies on product reformulation and reducing the serving sizes of menu items across countries and regions globally. This study utilized a scoping review, defined by Sucharew as a “research method and strategy to map, describe, and provide an overview of the published literature to identify relevant data and gaps to inform policymaking and research” [33]. The approach differs from a systematic evidence review that gathers, analyzes, and formally assesses the data to draw robust conclusions from the existing evidence for a well-defined issue. 

### 2.1. Scoping Review Step 1: Identify Restaurant Menu Labeling Policies

Step 1 of the scoping review was guided by the following research question: “What restaurant menu labeling policies have been implemented by countries across regions worldwide between January 2000 and February 2020?”. The lead investigator (S.R.G.P.) searched the WHO Global database on the Implementation of Nutrition Action (GINA) [34] and the World Cancer Research Fund International’s NOURISHING framework [35,36] for national, state, or municipal policies. Then, the data were screened, extracted, compiled and triangulated. The lead investigator used a cross-checked consultation process by reviewing the evidence with other relevant sources (i.e., governmental or health ministry websites and databases, international organizations, and governmental and nongovernmental agency reports) in English and Spanish.

### 2.2. Scoping Review—Step 2: Identify Evidence for Restaurant Menu Labeling Effects

Step 2 of the scoping review step was conducted using the five steps described by Arksey and O’Malley’s 2015 framework [37]. To enhance this methodology, we integrated scoping review recommendations by Levac et al. 2010 and Daudt et al. 2012 [38]. The process included identifying the research question, identifying relevant studies that met the inclusion criteria, study selection, charting the data, and summarizing the results. This research followed an iterative approach and used evidence and investigator triangulation to select and analyze the studies. 

#### 2.2.1. Identifying the Research Question 

The development of the research question was guided by the Population, Exposure, Outcome (PEO) framework that is widely used in qualitative social science or policy research rather than the PICO framework (i.e., population, intervention, comparison, and outcome) framework that is used to assess quantitative research outcomes [39,40,41]. This review defined population as transnational restaurants, including fast-food or QSR, FCR, and FSR chains; exposure was defined as voluntary and/or mandatory menu labeling policies, and the outcomes as food and beverage product reformulation and serving size reduction of restaurant menu items. Step 2 of the scoping review was guided by the following research question was: “What were the effects of voluntary and mandatory restaurant menu labeling policies on food reformulation and serving size available to restaurant consumers between January 2000 and February 2020?” 

#### 2.2.2. Identifying Relevant Studies 

The initial search was conducted using four electronic databases, including PubMed, CINAHL, Web of Science, and Google Scholar for peer-reviewed literature and gray literature. Only the first 100 hits sorted by relevance were considered for the Google Scholar database search. The databases were selected to be comprehensive and cover a broad range of disciplines, with guidance from a university research librarian. The PEO framework guided the identification of appropriate Medical Subject Headings (MeSH) terms and a combination of synonyms (Table 1; Table S1 provides MeSH terms definitions, and Table S2 provides the search details on each database). The reference sections of relevant articles were handsearched to identify further evidence not captured in the electronic database search. 

#### 2.2.3. Study Selection 

**Figure 1:**
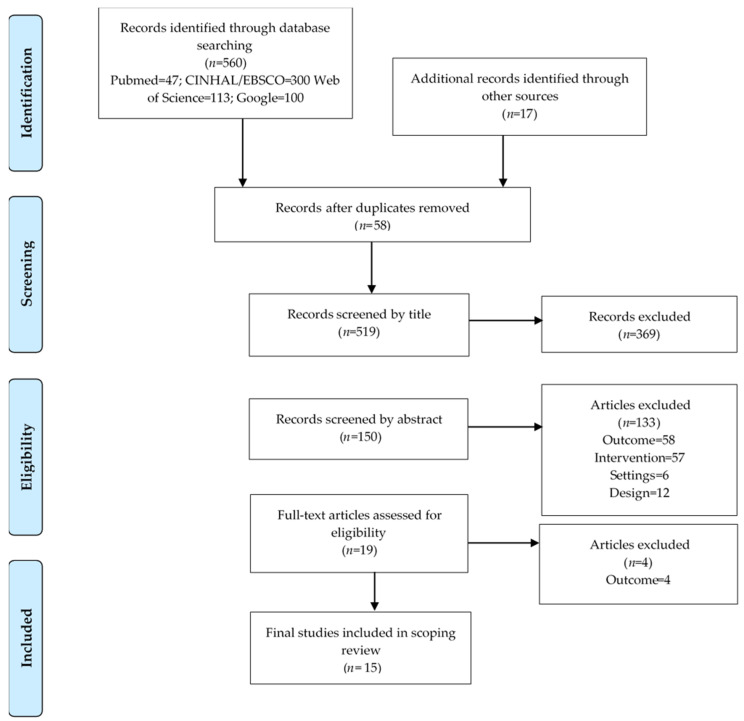
PRISMA flow diagram of the systematic study identification, screening, and selection of the studies for the scoping review.

The evidence selection was based on a priori inclusion and exclusion criteria. This scoping review was limited to peer-reviewed and gray literature published between 1 January 2000 and 29 February 2020 for English and Spanish-language studies and publications that explored the effect of menu labeling for restaurant chains that measured or evaluated the effects of menu labeling on product reformulation and serving size reductions. Studies were excluded for non-restaurant settings including cafeterias, laboratory settings, vending machines, schools, supermarkets, or independent food-retail establishments. Other evidence excluded was based on other outcomes related to consumers, purchase or consumption of nutrients, sales, pricing data, or described prevalence of business compliance. Literature reviews and studies based on packaged food labeling or other marketing strategies were considered to be different interventions and not included. Exclusion criteria also included literature reviews (i.e., scoping reviews, systematic reviews, and meta-analysis), which were removed and classified as the wrong type of study. All citations were imported into an EndNote X9 citation manager system and uploaded to the Covidence software, Cochrane’s primary screening and data extraction tool to support scoping and systematic reviews [42]. The screening process used the Preferred Reporting Items for Systematic Reviews and Meta-Analyses (PRISMA; Figure 1) guidelines that enabled the systematic searching, selection, and synthesis of the identified evidence [43]. The primary investigator (S.R.G.P.) removed duplicates, and a co-investigator (M.Z.) independently reviewed the title, abstract, and the full text of studies for inclusion against the eligibility criteria. A third co-investigator (V.I.K.) resolved any disagreements related to study inclusion.

#### 2.2.4. Charting the Data

From each selected study, two investigators (S.R.G.P. and M.Z.) extracted data on the author, year, country, study design, study purpose, sample, setting, data source, main outcomes, and disclosure of conflicts of interest. The data extraction was compiled in a single Microsoft Excel sheet. To assess the study quality, two investigators (S.R.G.P. and M.Z.) used the Johanna Briggs Institute’s critical appraisal eight-item checklist for analytical observational studies [44] and assigned a quality score ranging from poor, fair, or good. A third co-investigator (V.I.K.) was consulted to resolve any discrepancies to reach consensus through investigator triangulation. 

#### 2.2.5. Collating, Summarizing and Reporting Results 

We used a narrative synthesis [45] to report and summarize the evidence compiled for restaurant menu labeling policies related to the reformulation and serving size reductions of restaurant menu items, and to compare similarities, differences, and patterns among the evidence. A thematic analysis was also completed during the examination of the studies to identify topics and categorize the main results [46]. We disassembled the evidence to identify relevant themes based on the main outcomes. Thereafter, we reassembled the data across studies and organized it by positive effect if results showed a statistically significant *p*-value, no effect if results showed no statistically significant *p*-value or negative effects, and mixed-effects if results showed both findings for the effects of menu labeling.

## 3. Results

The search identified 3 voluntary and 8 mandatory menu labeling policies in 11 upper–middle and high-income countries defined by The World Bank classification. No policies were identified for low- or middle-income countries. Out of 577 screened studies, 15 studies met the inclusion criteria. Eleven studies were conducted in the Americas region (i.e., Canada and the US), two studies were conducted in the European region (i.e., the UK and Ireland), and two studies were conducted in the Western Pacific region (i.e., Australia) (Table 2).

### 3.1. Scoping Review Results for Step 1: Identify Restaurant Menu Labeling Policies

The implementation of voluntary or mandatory menu labeling policies has become popular throughout upper–middle and high-income countries of the world by region including the Americas *n* = 2, Europe *n* = 2, Eastern Mediterranean *n* = 3, Western Pacific *n* = 4; including Australia, Bahrain, Canada, Ireland, Malaysia, Saudi Arabia, South Korea, Taiwan, United Arab Emirates, the UK and the US (Table 3). No policies were found in the Africa and South-East Asian regions. 

We identified eight mandatory menu labeling policies across 11 countries. The US was the first country that enacted a mandatory national menu labeling law in 2010 that became effective on 1 May 2018 [61]. The Food and Drug Administration (FDA) has oversight for implementing the law and provided compliance guidance for industry. Section 4205 of the 2010 Affordable Care Act, Public Law 111-148 (HR 3590) mandated that restaurant chains and other retail establishments (i.e., convenience stores, coffee shops, grocery stores, cafeterias) with 20 or more US locations disclose calories on menus and menu boards and make other nutrition information available to customers upon request [61]. 

Several countries implemented a mandatory policy at national, state/provincial/territorial levels, including Australia [62], Canada [63], and the United Arab Emirates [64]. Between 2011 and 2018, the Australian government and Obesity Policy Coalition implemented various menu labeling schemes throughout four states and one territory. The current legislative schemes provide detailed requirements for chain food outlets, which include displaying the energy content in kilojoules for items on the menus, drive-through boards, tags, and other materials that display the name or price of products [62]. 

While mandatory policies have emerged, other countries have launched voluntary recommendations and guidelines to encourage restaurant chains and food industry businesses to display menu labeling for food and beverage items, which include Malaysia in 2008, followed by Bahrain in 2010, and the UK in 2011 [36]. These three countries are moving towards mandatory policies, and initiatives are being debated or incorporated into national plans. In 2016, the Malaysian government included the menu labeling strategy into its National Plan of Action for Nutrition 2016–2025, and plans to have a mandatory menu labeling policy by 2025 [65]. In 2018, Bahrain submitted a proposal to the Ministerial Cabinet that is currently under review for restaurants and cafes to voluntarily display calories [66]. Since 2015, mandatory menu labeling in Ireland has been under consideration and is now included in the National Obesity Policy and Action Plan 2016–2020 [67]. In 2011, the UK government released the voluntary policy for the Out of Home Calorie Labeling pledge as part of The Public Health Responsibility Deal, where businesses voluntarily committed to display the calorie content on menus [68]. The UK government is currently undertaking a consultation to implement menu labeling as a mandatory national policy [69,70,71].

All the policies across countries require the disclosure of energy content as calories or kilojoules. The US, Australia, and Dubai have mandatory policies that also require the display of daily energy intake statements so a customer can compare specific menu items to 2000 calories/day or 8700 kilojoules/day. Malaysia, Bahrain, and Korea expanded the nutrients that restaurants are required to report to include fat, protein, sodium, and added sugars. Taiwan is the only country that has a mandatory policy that requires the disclosure of caffeine and added sugars for beverages. 

### 3.2. Scoping Review Results for Step 2: Identify Evidence for Restaurant Menu Labeling Effects

The search yielded 560 articles across four electronic databases, and 17 additional records identified manually were included. After removing 58 duplicates, 519 records were screened. Of these, 369 records were excluded by title. Thereafter, 150 records were screened by abstract, 19 selected for full-text assessment, and 15 studies were included in the final scoping review (Figure 1).

Table 4 summarizes the studies that met all the inclusion criteria for the scoping review. Despite the search strategy including a wide range of years (from 2000 to 2020), all the included studies were published between 2012 and 2020, and more than half of the studies were published from 2018 to 2020. Eleven studies were conducted in the US, two in Australia, one in Canada, and one in the UK. A majority of studies (*n* = 14) were observational (i.e., longitudinal, case-control, and cross-sectional); and one study was a quasi-experimental design. The analyzed studies (*n* = 14) were conducted in diverse QSR, FCR and FSR chain settings, and a single study included convenience stores [55]. Diverse evidence sources were used across studies to assess the potential effects of menu labeling on food reformulation of food and beverage menu items and the serving reductions. Most of the studies used either the MenuStat Database (i.e., a free nutritional database provided by the New York City Department of Health and Mental Hygiene that provides nutritional information on menu items offered by the largest US chain restaurants; *n* = 7) or consulted business websites, visited establishments, or requested information via email and telephone (*n* = 7) to obtain nutrition content and serving size on menu items offered by restaurants chains. A single study for Canada used the Menu-FLIP database developed by the University of Toronto that provides nutrition data for chain restaurants [73]. The thematic analysis identified three main outcomes: (1) menu items, (2) the nutrition composition of menu items, and (3) newly introduced versus common or regular menu items. No conflicts of interest were found between the studies that could potentially influence the results. Table S3 shows the results of the study quality assessment. No studies were judged as being poor quality, four studies scored fair quality, and 11 studies were considered good quality. 

#### 3.2.1. Changes to Menu Items by Food and Beverage Category

The classification of menu items across studies varied. Most of the studies included appetizers and side dishes, main courses or entrees, and desserts. Six studies included children’s meals [49,50,52,56,59,74] and six studies examined beverages [19,48,49,50,53,57]. The evidence suggests that most of the changes made by restaurants were for appetizers and side dishes. Four studies showed statistically significant positive effects for calorie reduction [16,49,50,55], and two studies from the UK and the US reported mixed results [52,57]. 

Positive effects: Tran et al. (2019) conducted a study in the US during the period leading up to the federal menu labeling implementation date of May 2018 and found a reduction on calories mainly in entrees and dropping higher-calorie appetizers, sides, entrees, and desserts from the menus of pizzeria chains [55]. Bleich et al. (2017) described trends in calories from 19,391 US restaurant chain items that found differences in toppings: 93 kcal in 2008 to 84 kcal in 2015 (*p*-value for trend = 0.001) [50]. Bleich et al. (2016) found that calories declined between 2012 and 2014 for the main course items and children’s menu items at QSR, FCR, and FSR chains that suggested restaurants had voluntarily reduced calories in advance of the national menu labeling law [49]. Bruemmer et al. (2012) examined the calorie content of menu items in King County, Washington, and demonstrated statistically significant differences for the calorie content of entrees between 6 and 18 months of the menu labeling county law enactment. These results were presumably due to the reformulation of menu items for selected QSR and independent restaurant chains [16].

Mixed effects: A UK study assessed the effects of a national voluntary menu labeling guidelines for the top 100 UK chain restaurants ranked by sales [57]. Theis and Adams (2019) showed that while there was a reduction of calories and sodium for pizza, sandwiches, and toppings, baked goods items were higher in nutrients of concern (i.e., calories, fat, sugar, and sodium) in restaurants that provided menu labeling for customers [57]. Namba et al. (2013) found evidence that despite the increase in healthier entrees sold by US chain restaurants, a limited improvement was observed for the nutritional content of children’s entrees [52]. 

#### 3.2.2. Changes in the Nutritional Composition by Nutrients of Concern

The effects of menu labeling were measured by changes in the nutrition composition of menu items for four nutrients of concern, including calories (*n* = 14), sodium (*n* = 5), saturated fat (*n* = 3), and sugar (*n* = 3). A single US study from Washington state did not use these nutrients; rather the authors classified healthy versus unhealthy items based on 10 items examined by the Nutrition Environment Measures Surveys—Restaurant version (NEMSR) [54]. Two studies from the US and one from Canada assessed serving size reductions of menu items [16,47,54].

Positive effects: Bleich et al. (2020) reported the results of a longitudinal study (2012–2018) that examined nutrient trends for 28,238 food and beverage menu items from 28,238 US chain restaurants. The results found less calories in food items, and less calories and saturated fat in beverages, with results attributed to the US national menu labeling law [51]. Similar results were noted for six US studies that documented a significant decline in calories of certain items [19,48,49,50,53,55]. Besides energy, positive changes were reported for reducing the saturated fat and sodium content of menu items after the menu labeling implementation period in King County, Washington, that had more stringent menu labeling requirements before the national menu labeling law was passed in 2010 [16]. 

No effects: Two studies in Canada and Australia did not show significant results [47,58]. Saelens et al. (2012) reported that the availability of reduced portions actually decreased in King County, Washington, where menu labeling was implemented [54]. 

Mixed effects: Wu and Sturm (2014) assessed the energy and sodium changes from items offered by US chain restaurants after the national menu labeling law was passed in 2010 and in 2011. Results showed that QSR chains reduced the mean energy content of children’s menu entrees by 40 calories; however, upscale restaurants had increased the mean energy content of children’s menu entrees by 46 calories [56]. Similarly, Namba et al. (2013) examined the nutrient content of menu items after the national menu labeling law was passed in 2010 and in 2011, and found that the proportion of healthier menu items was higher in locations implementing restaurant labeling despite the mean calories of items that did not change [52]. Wellard-Cole et al. (2019) conducted a study in New South Wales, Australia, and found minimal decreases in energy, saturated fat, and sodium by specific QSR chains but and an increase in energy, sugars, and sodium from the QSR franchise called Hungry Jack’s (Burger King) [59]. 

#### 3.2.3. Newly Introduced Menu Items Versus Common or Regular Menu Items 

Seven studies conducted in the US and Canada [47,48,49,51,52,55,56] compared the differences between newly introduced menu items after the baseline year of the implementation of a menu labeling policy in 2018 with those that were dropped and/or stayed the same over the years. Five studies found positive effects [48,49,51,55,56], one study mixed effects [47], and one study found no effects [52]. 

Positive effects: Five US studies found significant changes made for newly introduced menu items that had fewer calories (from −57 kcal to −285 kcal) relative to popular menu items that were offered regularly and consistently at the chain restaurants [48,49,51,55,56]. 

No effects: Scourbutakos et al. (2019) investigated the early impact of the mandatory menu labeling law in the province of Ontario, Canada, that documented opposite results from the US studies that measured similar outcomes. The study found that newly introduced food items in 2017 contained more energy per serving compared with the newly introduced food items in 2016. The newly introduced menu items in 2017 also had significantly higher serving sizes compared with the newly introduced items from 2013 and 2016 [47]. 

Mixed effects: Namba et al. (2013) reported the results of a case-control study that examined five QSR chains that had voluntarily implemented menu labeling before the US national menu labeling law was passed in 2010. Three of the chains had improved the nutritional quality of items with healthier profiles of side dishes and children’s meals. However, two chains showed no reduction in calories of any menu items [52]. 

## 4. Discussion

This is the first comprehensive review published to document the number of countries that have enacted menu labeling policies, to compare the features of these policies, and to examine evaluations about the effect of menu labeling policies on the business practices of transnational restaurant chains globally. Step 1 of the scoping review identified 11 menu labeling policies or laws enacted by national, state or provincial, and/or municipal authorities in upper–middle and high-income countries between 2010 and 2020. The governments in eight countries had enacted mandatory policies (i.e., Australia, Canada, Ireland, Saudi Arabia, South Korea, Taiwan, United Arab Emirates, and the US). The governments in three countries had enacted voluntary policies (i.e., Bahrain, Malaysia, and the UK). Step 2 of the scoping review summarizes the results and evidence gaps from 15 published studies (2012 to 2020) on existing menu labeling policies across four countries (i.e., Australia, Canada, the UK, and the US). Overall, the studies found mixed results, and only the US studies showed positive effects of restaurant menu labeling policies to reformulate items or introduce new healthier items ranging from 57calories to 285 fewer calories/item. Studies conducted in Australia, Canada, and the UK found either no effect or mixed effects of menu labeling policies on businesses to reformulate or introduce new menu offerings.

Step 2 of the scoping review revealed a major lack of published evidence for the effects of menu labeling on restaurant business for other regions of the world that have policies in place identified in step 1 (Table 2). No studies were found on the effects of menu labeling policies on restaurant food reformulation and serving sizes in the Asian region (i.e., Malaysia, South Korea, and Taiwan); Middle East region (i.e., Bahrain, Dubai, Saudi Arabia, and the United Arab Emirates); and European region (i.e., Ireland). This may have been due to no evaluations conducted, evaluations that were not available in the public domain, or published in languages other than English or Spanish.

The mandatory restaurant menu labeling policy compliance rate for disclosing energy (calories or kilojoules) was high in the US (94% after May 2018) [75] and in New South Wales, Australia (95%) [76]. However, subsequent evaluations in New South Wales showed that this compliance had not translated into restaurants making significant reductions in energy for menu items by 2016 [59]. A 2018 evaluation of restaurant menu labeling compliance across four Australian states (including New South Wales) and one territory showed high menu labeling compliance reported by 11 chain restaurants [77]. However, independent evaluations are needed to verify industry-reported results. 

The menu labeling policies reviewed were found across upper–middle and high-income countries. However, the existing evidence highlights that eating away from home is increasing among populations creating room for menu labeling policies. The 2015 Nielsen Global Out-of-Home Dining Survey conducted among more than 30,000 adults in 61 countries found that about half of respondents (48%) reported eating out one or more times weekly (REF). Consumers (22%–26%) in the Asia-Pacific region (i.e., Hong Kong, Taiwan, Malaysia and Thailand) reported eating out daily, and other countries with menu labeling legislation (i.e., Saudi Arabia and the US) reported rates of eating away from home daily (12%–15%) that exceeded the global average of 9 percent [10]. The survey found that three out of the top five countries with the highest percentage of respondents that eat lunch away from home are in Latin America: Chile, Brazil, and Colombia [10]. Popkin and Reardon (2018) confirmed that since 1995, people are increasingly spending more of their income on eating out of home, with higher significant increases in Brazil, Chile, and Colombia [78]. A Nielsen Global Survey of food labeling trends among 25,000 consumers in 56 countries found that 80 percent of respondents expressed that fast-food restaurants should include calorie labeling and other nutrition information either sometimes or always, and, support was strongest in Latin America, North America and Europe [79]. Given these trends, there is a need to evaluate menu labeling policies of countries in these regions.

The small number of studies that assessed other nutrients of concern (i.e., saturated fats, trans fats, sodium, and added sugars) [16,51,56,57,59] rather than just energy might be the consequence of policies limiting the regulation to reporting the energy content. All 11 countries that have implemented restaurant menu labeling policies require the disclosure of energy (i.e., calories or kilojoules). Only three countries (i.e., Australia, United Arab Emirates, and the US) require contextual information to display the daily energy intake recommended for the average adult (i.e., 2000 calories/day or 8700 kilojoules/day). Of these three countries, no evaluation was available for Dubai, and only two published evaluations were available for New South Wales, Australia, that found no significant effects. Results showed that two voluntary policies (Malaysia and Bahrain) and one mandatory policy (South Korea) included disclosure of fat, protein, sodium, and sugar besides calories. However, no evaluations were available to assess industry changes to reduce the availability of nutrients of concern (i.e., sodium, saturated fat, trans fat, and added sugar) linked to obesity, and diet-related NCDs have not been assessed yet. 

It is important to note that the US studies showed a positive effect of menu labeling on restaurants to reduce calories for newly introduced items, especially appetizers and side dishes, may have been related to a longer time frame between the legislation enactment in 2010 and the published studies with positive effects (2015–2020) [16,19,48,49,50,51,53,55]. It is possible that the US restaurant sector had a longer period of time to implement changes that complied with the national law. Two US studies showed mixed results where the time factor could have influenced. Namba et al. (2013) evaluated the effect of menu labeling on QSR chain menus from 2005 through 2011, and most of the assessed years were before the national menu labeling law was passed [52]. In contrast, the menu labeling legislation passed in 2015 in Ontario, Canada, was implemented in January 2017. The Canadian study showed baseline data (2010–2016) no effects of menu labeling on the chain restaurants reformulating to offer healthier items [47]. Australia initiated mandatory menu labeling legislation in New South Wales in 2011, which was expanded to the Australian Canberra Territory and three states, including Victoria, which enacted mandatory menu labeling in 2018. The studies conducted in Australia showed both mixed [59] and no effect [58] of food reformulation or serving size reductions. 

The type of policy might have influenced the study outcomes besides the time factor. The UK implemented a voluntary menu labeling policy that could have played a role in the mixed-effects found by Theis and Adams 2019 [57]. Several challenges are associated with mandatory policies enacted at the state or territorial levels (Australia) or the provincial level (Canada) that may lead to inconsistencies in legislation between jurisdictions and across the outlet threshold (chain versus non-chain), variations in the provision of voluntary, readable and standardized nutritional information to customers, and inability to customize menu ordering [77]. 

The study design may also explain the results from this review since the studies showing positive effects in the US were observational and longitudinal. The availability of longitudinal data from the MenuStat database could justify why the US studies showed positive effects for national menu labeling over eight years (2010–2018) compared to other countries that had a shorter time frame from the enactment of legislation. Experimental, quasi-experimental, and observational, case-control studies that compared non-regulated periods or jurisdiction versus regulated ones found no or mixed effects, respectively. This may indicate that industry changes may have happened for other reasons and/or policies independently from the menu labeling policy. In addition, studies that found positive effects have analyzed changes among items, and those that assessed effects among menus instead, have found no or mixed effects. These findings suggest that industry may have introduced positive changes to some items but kept the overall nutritional quality of the menu as a whole unchanged. The majority of study designs from the reviewed articles were observational, which cannot determine causation, and reverse causality needs to be explored. Restaurants could have changed their products before implementing menu labeling, or food businesses and non-restaurant businesses could have adopted pledges and commitments on items that are often offered in restaurants. Some recent US voluntary initiatives to improve the nutritional content of food and beverage products are the Healthy Weight Commitment [80] and the Children’s Food and Beverage Advertising Initiative [81] in the US. 

A robust body of evidence has shown that food reformulation may reduce or eliminate sodium and trans fats, both of which are identified by the WHO as a cost-effective strategy used across different countries to improve diet quality and reduce obesity and diet-related NCD risks [82,83,84,85,86,87,88,89,90]. Food and beverage product reformulation may have a greater impact on the entire population than strategies that encourage healthy choices that may or may not influence consumer behavior change because the decline in energy (calories or kilojoules) is distributed across populations that frequently consume the modified products [88,91,92]. 

Our scoping review results identified several challenges. First, evaluations were published for only four of 11 countries that had passed legislation between 2010 and 2020. This suggests that policymakers are not investing adequate resources to monitor and evaluate the effects of menu labeling policies. Second, only the US studies that evaluated the effects of a mandatory national policy showed that restaurants had reduced calories for some newly introduced menu item categories, but did not reduce calories or the serving sizes of popular items frequently consumed. This is a challenge because expert bodies have recommended nutrient targets for menu item categories that are not being used as reference points to evaluate industry progress [21]. Third, while the WHO has recommended nutrition labeling and reducing portion sizes as strategies to reduce energy intake, our study found no evidence to support menu labeling legislation as a cost-effectiveness “best buy” strategy to reduce NCD-related disability and mortality in low- and middle-income countries [14].

### 4.1. Implications for Policy, Practice, and Research 

Our results suggest that menu labeling legislation in the absence of other supportive strategies is unlikely to produce a meaningful change among restaurant practices to expand healthy menu items for all customers. Menu labeling is one of eight marketing-mix and choice architecture strategies that restaurant businesses can use to nudge customers toward healthy food environments 20 [93,94]. A compelling business case must be made to persuade chain restaurants to adopt these strategies to improve their corporate image and attract new customers who want healthy meals [95].

Table 5 suggests several actions for stakeholders, including governments, the WHO, restaurant businesses, private foundations, researchers, and civil society organizations to develop, implement, and evaluate comprehensive restaurant menu labeling policies. 

Government action is needed to implement national comprehensive menu labeling policies to have a significant effect on food reformulation and serving size reduction. Evidence still needs to be stronger to confirm these positive effects, and it is clear that voluntary efforts by industry are not enough. Only one study [57], based on the UK voluntary policy, discussed that food business initiatives and goodwill are insufficient for restaurant menu labeling to become a cost-effective strategy to address obesity and diet-related NCDs. Littlewood et al. (2016) have suggested that restaurants are more likely to improve their performance to offer healthier options with mandatory government requirements [25]. 

Digital technologies (i.e., online ordering through apps and digital menu boards) are being used more frequently to reach more customers that may either support or undermine the positive effect of menu labeling. The coronavirus or COVID-19 pandemic has created a new trend where restaurant businesses have moved to digital online and delivery, in response to the economic crisis that the pandemic has caused worldwide. Future policies and research should examine how restaurants change menu items based on customers’ online ordering experience, use of onsite digital technology computerized touch screens and smartphones, use of algorithmic nudging to influence customers’ choices, and how customers use digital technologies available through third-party delivery apps and businesses such as UberEats and DoorDash. Research could also examine how to leverage digital technology to encourage menu item reformulation and serving size reductions while encouraging customers to purchase the healthiest menu items [96]. 

Effective policy actions require regulatory oversight to ensure accountability [97]. The engagement of diverse sectors will help to strengthen the accountability process. Civil society organizations should mobilize efforts to support restaurant menu labeling initiatives and can perform independent evaluations that are shared with industry actors and government regulatory bodies. It is common for the industry sector to oppose these initiatives based on evidence from Ireland [67] and in the UK [69], where national menu labeling has been under consideration by Congress since 2015. 

This research adds to the literature by identifying the knowledge gaps about the effects of restaurant and fast-food chain menu labeling on food reformulation and serving size reductions. Further research is needed to assess ongoing restaurant menu labeling policies from the Americas region (especially Latin and Central American countries), European, Eastern Mediterranean, African and Western Pacific regions for the short-term, mid-term, and long-term effects. More research is needed to explore whether restaurant menu labeling can reduce serving sizes of menu items in middle-and low-income countries. Experimental studies are needed to explore reverse causation and whether restaurant menu labeling policies will be effective in different countries by context. Finally, the WHO has clearly stated that obesity and NCDs are risk factors for COVID-19 [98]. Governments are implementing “new guidelines” for re-opening business and reset the economy and should prioritize in their political agenda policies that encourage healthier food environments to ensure that nutritious food is available for all populations as an integral strategy. 

### 4.2. Study Strengths and Limitations 

This scoping review has several limitations common to the nature of the study (i.e., map, describe, and provide an overview of the published literature to identify relevant data to inform policymaking and research). The exploratory scope of this review does not enable conclusions about the topic. However, these results may provide valuable insights for research and policy actions, especially regarding the monitoring and evaluation of implemented policies within and across countries to rigorously understand whether and under what conditions menu labeling could have an effect on restaurant businesses. It is possible that the use of additional literature databases would have yielded further articles. Given the involvement of an expert librarian, it was anticipated that the selected databases were appropriate to capture the breadth of research on this topic. In addition, this review also assessed the quality of the selected studies. We limited the search date to 2000. No studies that met the inclusion criteria were found between 2000 and 2011; therefore, we believe that our search captured the majority of relevant articles for the topic. Literature in other languages than English and Spanish were excluded, so research for countries that had legislation and evaluations published in other languages may have been missed. Lastly, all the selected studies were conducted in high-income countries; therefore, these results cannot be generalized to middle- or low-income country settings. 

## 5. Conclusions

The trend of increased eating away from home across countries is a call for mandatory menu labeling policies to improve healthy offerings to support a healthy diet worldwide. The overall evidence from this review is mixed on the effect of menu labeling policies for transnational restaurants and fast-food chains on food reformulation. The positive effects were from observational and longitudinal studies conducted within the period the legislation was enacted in the US and mainly for food reformulation of the energy content of menu items, and the introduction of new healthier menu items, not for overall changes among the menus. Case-control and quasi-experimental studies found no or mixed effects. Considerable gaps in the evidence remain, particularly regarding the effects of the implemented policies across regions at mid- and long-term, research in middle- and low-income countries, and reverse causation of restaurant menu labeling policies. Moreover, while all the enacted policies across countries request to display energy content, additional nutrients of concern could be included to have a greater impact. These results may inform governments, civil society, academics, and the restaurant industry to develop comprehensive and robust restaurant menu labeling policies that promote healthy dietary choices to reduce obesity and NCD risks worldwide. 

## Figures and Tables

**Table 1:** Systematic search strategy for the scoping review.

PEO Framework	MeSH Terms and Synonyms
Population	“Restaurants”[MeSH] OR “Food Services”[MeSH] OR “Food Supply”[MeSH] OR “Fast Foods”[MeSH] OR “Food Industry”[MeSH] OR “Food-Processing Industry”[MeSH] “Chain restaurant*” OR restaurant or “food retail” OR “food services*” OR “food supply” OR “food supplies” OR “fast food*” NOT Schools [MeSH] AND
Exposure	“Policy”[MeSH] OR “Nutrition Policy”[MeSH] OR “Public Policy”[MeSH] OR “Health Policy”[MeSH] OR “Government Regulation”[MeSH] OR “Legislation” [Publication Type] OR “Legislation, Food”[MeSH] OR “Voluntary Programs”[MeSH] OR “Mandatory Programs”[MeSH] OR “Patient Protection and Affordable Care Act”[MeSH] OR “Mandatory Policy” OR “Voluntary Policy” OR “Self-regulation” OR “Nutrition policies” OR Guideline OR “Food Policy” AND “Food Labeling”[MeSH] OR “Product Labeling”[MeSH] OR “Food product label*” OR “Menu label*” OR “Restaurant label*” OR “Restaurant label” OR “Restaurant menu label*” OR “Food calories” OR “Nutrient label*” OR “Food content” NOT “Food Packaging”
Outcome	“Food”[MeSH] OR “Beverages”[MeSH] OR “Food and Beverages”[MeSH] OR “Food Ingredients”[MeSH] OR “food product*” OR “Fast food” AND “Food Quality”[MeSH] OR “Food, Formulated”[MeSH] OR “Serving Size”[MeSH] OR “Portion Size”[MeSH] OR “Food reformulation” OR “Reduce* Portion*” OR “Reduce* size*” OR “Product reformulation”

PEO framework: (P) Population—transnational restaurants; (E) Exposure—voluntary and mandatory policies; (O) Outcome—food reformulation and serving size reductions.

**Table 2:** Two-step scoping review results across countries by world region*.

* World Region	Scoping Review—Step 1	Scoping Review—Step 2
	Identify Policies (Policies = 11)	Identify Evidence (Studies *n* = 15)
Africa	None identified (*n* = 0)	None identified
Americas (*n* = 2)	Canada and US (*n* = 2)	Canada (*n* = 1): Scourboutakos et al. (2019) [47]. US (*n* = 11): Bleich et al. (2015) [48], Bleich et al. (2016) [49], Bleich et al. (2017) [50], Bleich et al. (2018) [19], Bleich et al. (2020) [51], Bruemmer et al. (2012) [16], Namba et al. (2013) [52], Petimar et al. (2019) [53], Saelens et al. (2012) [54], Tran et al. (2019) [55], Wu et al. (2014) [56]
South-East Asia	None identified (*n* = 0)	None identified
Europe	Ireland and the UK (*n* = 2)	UK (*n* = 1): Theis et al. (2019) [57]
Eastern Mediterranean	Bahrain, Saudi Arabia, United Arab Emirates (*n* = 3)	None identified
Western Pacific	Australia, Malaysia, South Korea, Taiwan (*n* = 4)	Australia (*n* = 2): Wellard-Cole et al. (2018) [58], Wellard-Cole et al. (2019) [59].

US: United States; UK: United Kingdom. * World Health Organization regional groups [60].

**Table 3:** Implemented menu labeling policies across countries worldwide, 2008–2020*.

Country, Year	Policy Type	Action
Australia, 2011–2018	Mandatory, four states and one territory	Restaurant chains with ≥20 outlets in the state, or 50 or more across the country, are required to present the energy content (kilojoules) and include a daily intake statement on menus and menu boards. Similar food businesses are invited to voluntarily implement menu labeling.
		States of New South Wales, 2011: Food regulation 2011
		Australia Capital Territory, 2012: Amendments to Food Regulation 2002
		Australia, South Australia, 2012: Amendments to Food Regulation 2002
		Australia, Queensland, 2017: Amendments to Food Act 2006
		Australia, Victoria, 2018: Amendment to Food Act 1984
Bahrain, 2010	Voluntary, national	The Nutrition Section of the Ministry of Health recommends that fast-food chain restaurants display nutrients per serving, including calories, fat, protein, carbohydrates, salt/sodium, and sugar.
Canada, Ontario, 2017	Mandatory, province	In 2015, Ontario’s Healthy Menu Choices Act, part of the Making Healthier Choices Act (Bill 45) in the Ontario Regulation 50/16, requires food service establishments with 20 or more businesses to depict calories for menu items on paper and electronic menus, menu boards, drive-through menus, menu applications, and advertisements or promotional flyers.
Ireland, 2015	Mandatory, national	In 2015, the Health Service Executive approved the implementation of Calorie Posting Policy across health services in all food and beverage facilities (i.e., restaurants, coffee shops, catering services, and vending machines).
Malaysia, 2008	Voluntary, national	In 2008, the Malaysian government released voluntary guidelines for the advertising and nutrition labeling of restaurant chains to display nutrient information on the menu items (i.e., calories, carbohydrates, protein, fat, and sodium for food and total sugar for beverages).
Saudi Arabia, 2018	Mandatory, national	In 2018, the Saudi Food and Drug Authority launched mandatory measures that require calorie labeling on menu items for all food facilities, including cashier desks, menu boards, table menus, drive-through menus, phone, and web applications.
South Korea, 2010	Mandatory, national	In 2010, the South Korean government enforced through the Special Act on Safety Control of Children’s Dietary Life that restaurants with more than 100 outlets are required to report energy, total sugars, protein, saturated fat and sodium on the menus
Taiwan, 2015	Mandatory, national	From 2015, the Taiwanese Act Governing Food Safety and Sanitation that regulates business chains (i.e., convenience stores, drink vendors, and fast-food restaurants) requires the labeling of the sugar and caffeine content of prepared-when-ordered drinks.
United Arab Emirates, 2020	Mandatory, state/emirate	The 2017–2020 National Nutrition Agenda for Dubai requires food retailers to display the calorie content of menu items and a daily intake statement, effective 1 January 2020.
United Kingdom, 2011	Voluntary, national	From 2011–2015, the Out of Home Calorie Labelling pledge, part of the government’s Responsibility Deal (2010 to 2015), established for businesses with 45 or more food establishments the need to provide calorie information on menus in England, Scotland, and Wales. In 2012, the Food Standards Agency worked with Northern Ireland and the local food industry to encourage calorie labeling on menus
United States, 2010-2018	Mandatory, national	In 2010, Section 4205 of the Affordable Care Act, Public Law 111-148 (HR 3590), mandated that restaurant chains and other food retail establishments (i.e., convenience stores, coffee shops, grocery stores, cafeterias) with 20 or more locations would be required to disclose calories and daily intake statements on menus and menu boards and make other nutrition information available to customers upon request. The law became effective on 1 May 2018.

* Policy is defined as a law, procedure, regulation, rule, or standard that guides how government, businesses, and organizations operate and how citizens live their lives [72].

**Table 4:** Summary of articles included in the scoping review.

Author Year	Country	Study Design	Purpose	Sample	Setting	Data Sources	Menu Items	Nutrition Composition	New vs. Common Menu Items	Effect *
Bleich et al. 2015 [48]	USA	Observational, longitudinal	Compare differences in calorie counts from menu labeling, 2012–2014	23,066 menu items from 66	Restaurant chains	MenuStat	Food and beverages	Calories. Average per item calories restaurants with voluntary labeling was significantly lower than those without the labeling (−286 kcal: 232 vs. 519)	Lower calorie content for new menu items introduced in 2013 (−182 kcal: 263 vs. 445; and in 2014 (−110 kcal: 309 vs. 419)	Positive
Bleich et al. 2016 [49]	USA	Observational, longitudinal	Describe trends in calories available in US chain restaurants from 2012 to 2014 to better understand restaurant-driven changes	23, 066 menu items over 3 years in 66 large chain restaurants	QSR, FCR and FSR chains	MenuStat	Appetizers and sides, main courses, desserts, toppings, beverages, and children’s menu items. New food, beverages, and children’s menu items all had fewer mean calories relative to old menu items (66, 47, 43, and 35 fewer calories, respectively)	Calories. Predicted mean per-item calories in new main course items in 2013 had 85 fewer calories relative to old main course items in 2012. Calories declined in pizza (−120 calories), sandwiches (−82 calories), and salads (−68 calories)	Menu items newly introduced in 2013 and 2014 had significantly fewer calories relative to items on the menu in 2012 (2012 vs. 2013: −71 calories; 2012 vs. 2014, −69 calories)	Positive
Bleich et al. 2017 [50]	USA	Observational, longitudinal	Understand trends in calories in chain restaurants before and after the passage of the menu labeling rule	19,391 menu items from chain restaurants	QSR, FCR and FSR chains	MenuStat	Appetizers and sides, fried potatoes, main courses, toppings, beverages, and children’s menu items. Largest differences were found for toppings that reduced from 93 kcal in 2008 to 84 kcal in 2015	Calories. Overall calories declined from 327 kcal in 2008 to 318 kcal in 2015	-	Positive
Bleich et al. 2018 [19]	USA	Observational, longitudinal	Compare mean calories for items that remained on restaurant menus with items dropped from the menu	27,238 menu items from restaurant chains	Restaurant chains	MenuStat	Appetizers and sides, main courses, desserts, and beverages	Calories. Items that were dropped had 71 more calories	Items that stayed on the menu in all years had fewer calories than those items that were dropped (448 calories vs. 733 calories)	Positive
Bleich et al. 2020 [51]	USA	Observational, longitudinal	Update calorie and nutrient trends 2012–2018 of menu items across restaurants	28,238 menu items from chain restaurants	Fast-food, fast-casual, and full-service restaurant chains	MenuStat	Appetizers and sides, main courses, fried potatoes, desserts and baked goods	Calories, saturated fat, sodium, sugar and protein.Significant changes in food (sugar −0.67 g) and beverages (unsaturated fat −1.8 g, protein −2.7 g). Trend in years: calories −120 kcals (−25%), saturated fat −3.4 g (−41%), unsaturated fat −4.5 g (−37%), non-sugar carbohydrates −10.3 g (−40%), and protein −4.3 g (−25%)	Significant changes were found among all newly introduced items. It is possible that the declines in calories and nutrients in this study are related to local or national nutrition policies	Positive
Bruemmer et al. 2012 [16]	USA	Observational, longitudinal	Evaluated changes in energy, saturated fat, and sodium content of entrees 6 and 18 months that occurred following the implementation of menu labeling regulation	37 chains	QSR and FSR chains	Personnel visited and recorded energy content from menu labels, and websites	Entrée items. Calorie content decline in overall average entrée calories (41 fewer calories post labeling; 73 fewer calories at full-service restaurants and 19 fewer at QSR) when comparing 6 and 18 months post labeling	Calories and sodium. Decrease in energy, saturated fat, and sodium content between the two study periods following implementation of menu regulation for menu items that were present at both time periods. Saturated fat and sodium levels decreased significantly across all chains and SD chains	-	Positive
Namba et al. 2013 [52]	USA	Observational, case-control	Evaluate the effect of menu labeling on menu offerings over 7 years, from 2005 through 2011	3887 menu items from chain restaurants	Top 50 QSR chains	Restaurant websites	Entrées, sides, and children’s entrées. Case restaurants increased the proportion of healthier entrées after labeling regulations: from 13% during years 2005 through 2008, up to 20% by 2011 with a mean difference of 5% pre–post 2008 in cases relative to controls. The prevalence of healthier side dishes was higher among case restaurants than controls (23% vs. 15%, respectively). Healthier children’s entrées at case restaurants were higher	Calories. Regression models found no statistically significant changes over time in nutrient averages and no statistically significant differences between the nutritional averages of case and control restaurants	3 of 5 labeled restaurants improved their offerings. Control restaurants had a lower proportion of healthier items than cases. 2 of 5 showed no improvement and even launched new options, that increased average calories by over 20% and cholesterol by almost 140%	Mixed
Petimar et al. 2019 [53]	USA	Observational, longitudinal	Evaluate calorie labeling in mean calories purchased, pre-2015–2017 and post menu labeling implementation period 2017–2018	59 restaurants	Restaurants	Menustat	Entrées, sides, sugar-sweetened beverages	Calories. The top 50 menu offerings purchased in 2017–18 had a median of 350 calories (interquartile range 440–760) pre-implementation and a median of 340 calories (440–760) post-implementation.	-	Positive
Saelens et al. 2012 [54]	USA	Experimental, quasi-experimental	Examine changes in restaurants from before to after nutrition-labeling regulation in a regulated versus a nonregulated county of Washington state	Top 10 QSR chains	QSR and independent restaurant chains	Nutrition Environment Measures Survey Restaurant (NEMS-R)	Healthy vs. Unhealthy based on 10 items examined by the Nutrition Environment Measures Surveys—Restaurant version (NEMS-R)	The healthfulness of children’s menus improved modestly over time, but not differentially by county. Availability of reduced portions decreased in the regulated county	-	No effect
Scourboutakos et al. 2019 [47]	Canada	Observational, longitudinal	Investigate the early impact of Canada’s mandatory menu labeling legislation on calorie levels in foods offered on chain restaurant menus before, leading up to, and at the point-of-implementation, 2010 - 2017	2988 foods sold by 28 restaurant chains	QSR and FSR chains	Menu-FLIP database	Entrées, pizza, breakfast foods, side dishes, baked goods/desserts, kids’ foods	Calories. The average calories per serving on restaurant menus increased from 306 (SD = 6) kcal to 346 (SD = 6) kcal, between 2010 and 2017. An increase in serving size, from 155 (SD = 3) to 172 (SD = 3) grams, between 2010 and 2017. Calorie density (kcal per 100 g) did not significantly differ between 2010 and 2017. Significant increase in serving sizes among sit-down restaurants of 12 g per serving between 2010 and 2017	Overall, new foods introduced in 2017 were significantly higher in calories per serving compared with those introduced in 2016. New foods introduced in 2017 had significantly higher serving sizes compared with new foods in 2013 and 2016	No effect
Theis et al. 2019 [57]	UK	Observational, cross-sectional	Determine whether there are differences in the energy and nutritional content of menu items served by UK restaurants vs. without voluntary menu labeling	100 UK chain restaurants	QSR and FSR chains ranked by sales	Restaurant websites	Appetizers and sides, baked goods, beverages, burgers, desserts, fried potatoes, mains, pizza, salads, sandwiches, soup, toppings, and ingredients. Main dishes (i.e., pizza and sandwich) had less sugar and salt. Toppings and ingredients had less fat and protein than items from restaurants without menu labeling. Baked goods items from restaurants with menu labeling had, more energy, fat, saturated fat, sugar but protein and more salt	Calories, saturated fat, sodium, sugar, carbohydrates, and protein. Restaurants with menu labeling had 45% less fat (beta coefficient 0.55; 95% CI 0.32 to 0.96) and 60% less salt (beta coefficient 0.40; 95% CI 0.18 to 0.92)	-	Mixed
Tran et al. 2019 [55]	USA	Observational, longitudinal	Describe trends in calories among food items sold in US convenience stores and pizza restaurant chains from 2013 to 2017	1522 food items from convenience stores and 2085 items from pizza restaurant chains	Pizza restaurant chains	MenuStat	Appetizers and sides, main courses, and desserts. Lower calories among items that stayed on the menu compared to items dropped (overall: −60 kcal; appetizers and sides: −200 kcal *p* < 0.001; main courses: −50 kcal *p* = 0.03; desserts −60 kcal)	Calories. Reduced calories in menu items (−56 kcal: 390 kcal in 2013 vs. 334 kcal in 2017), appetizers (−230 kcal: 367 kcal in 2013 vs. 137 kcal in 2017)	Calories were lower among items that stayed on the menu compared to items dropped. Lower-calorie pizza options were introduced, but no significant changes	Positive
Wellard-Cole et al. 2018 [58]	Australia	Observational, longitudinal	Examine the energy content of Australian fast-food menu items before and after menu board labeling	522 menu items from fast-food chains	5 of the largest Australian QSR chains	Fast-food websites	Breakfast, burgers, desserts, chicken and seafood, salads, sides, sandwiches and wraps	Calories. No differences in energy per serving items, content per 100 g for burgers was higher after implementation (1040 vs. 999 kJ/100 g before implementation,)		No effect
Wellard-Cole et al. 2019 [59]	Australia	Observational, longitudinal	Investigate the nutrient composition of children’s meals offers at fast-food chains, compare with children’s daily requirements and recommendations and determine if results have changed prior to the implementation of menu labeling	289 children’s meals	Australian QSR and FCR chains	Fast-food websites, email and telephone requests, and personnel visits	Children’s meals per restaurant chain	Calories, saturated fat, sodium and sugar. Minimal changes were found. Meals from Chicken Treat reduced mean energy (−600 kJ/serving), saturated fat (−9·4 g/serving) and Na (−121 mg/serving), and from Red Rooster (−410 kJ/serving) and sugars (−11·8 g/serving), KFC reduced saturated fat (−10·5 g/serving). However, meals from Hungry Jack’s increased in energy (345 kJ/serving), sugars (8·6 g/serving), and Na (187 mg/serving)	-	Mixed
Wu et al. 2014 [56]	USA	Observational, longitudinal	Track changes in the energy and sodium content of US chain restaurant main entrées between spring 2010 (when the Affordable Care Act was passed) and spring 2011	25,256 regular menu entrées from 213 restaurant brands	Top US chain restaurants based on 2008 sales	Restaurant websites, and email request	Regular menu entrées and children’s menu entrées	Calories and sodium. 26 restaurants reduced sodium in newly added items by 707 mg on average. Significant decrease in mean energy (−40 kcal. Two upscale restaurants with children’s menu entrées had a significant increase in mean energy (46 kcal). Items removed from children’s menus were 36 kcal lower	Higher-sodium items decreased by 70 mg (*p* = 0.027) in added vs. removed items on regular menus. Calories decreased by 57 kcal (*p* = 0.047) for added vs. removed children’s entrées	Mixed

* Effect: positive (if results showed a statistically significant *p*-value), no effect (if results showed no statistically significant *p*-value or negative effects), and mixed-effects (if results showed both) on menu labeling. QSR: quick-service restaurants; FCR: fast-casual restaurants; FSR: full-service restaurant. kJ: kilojoules.

**Table 5:** Recommended actions for stakeholders to develop, implement, and evaluate comprehensive restaurant menu labeling policies.

Food System Actors	Recommended Actions
Governments	Provide enough support for food service restaurant businesses to facilitate a low-cost, sustainable, and accountable policy. Policies could be improved to incentivize more holistic menu changes by requiring the display of energy and other nutrients of concern, including fats, sodium, and added sugars for each item offered by restaurants.
World Health Organization	Issue recommendations for governments and transnational restaurants and their franchise businesses, and food service providers to harmonize, standardize, and apply a universal set of healthy dietary standards across countries and regions.
Restaurant businesses	Make commitments and increase transparency to meet product profile targets based on WHO- or expert-recommended guidelines
Private foundations	Provide technical assistance and incentivize transnational restaurant chains to implement, monitor, and evaluate menu labeling policies across countries and regions.
Researchers	Expand external monitoring and evaluation efforts of transnational restaurant chains to assess their compliance with WHO- or expert-recommended guidelines across countries and regions. Examine how digital technology could be used to leverage the effects of restaurant menu labeling policies.
Civil society organizations	Use social media advocacy, public awareness campaigns, and shareholder resolutions to encourage governments to implement comprehensive restaurant menu labeling policies for healthy product reformulation and portion size reduction for products sold to customers across countries and regions.

## Supplementary Materials

The following are available online at https://www.mdpi.com/2072-6643/12/6/1544/s1. Table S1: MeSH terms definitions. Table S2: Search details on each database. Table S3: Quality assessment results based on the Johanna Briggs Institute critical appraisal checklist.
