# Supplementary material for: Effects of Menu Labeling Policies on Transnational Restaurant Chains to Promote a Healthy Diet: A Scoping Review to Inform Policy and Research
Source: Nutrients. 2020 May 26;12(6):1544. doi: 10.3390/nu12061544 (PMC7352298; doi:10.3390/nu12061544)
Supplement: Supplementary file 1 [file nutrients-12-01544-s001.zip › Supplementary material/Supplementary material 1.docx]

**Supplementary material 1.** MeSH terms definitions from PubMed U.S. National Library of Medicine

| **PEO framework** | **MeSH Term** | **Definitions** |
| --- | --- | --- |
| **Population** | *Restaurants* | An establishment where meals are cooked, sold and served to customers. |
|  | *Food Industry* | The industry concerned with processing, preparing, preserving, distributing, and serving of foods and beverages. |
|  | *Food-processing industry* | The productive enterprises concerned with food processing. |
|  | *Food services* | Functions, equipment, and facilities concerned with the preparation and distribution of ready-to-eat food. |
|  | *Food supply* | The production and movement of food items from point of origin to use or consumption. |
|  | *Fast foods* | Prepared food that is ready to eat or partially prepared food that has a final preparation time of a few minutes or less. |
|  | *Schools* | Educational institutions |
| **Exposure** | *Policy* | A course or method of action selected to guide and determine present and future decisions. |
|  | *Public Policy* | A course or method of action selected, usually by a government, from among alternatives to guide and determine present and future decisions. |
|  | *Health Policy* | Decisions, usually developed by government policymakers, for determining present and future objectives pertaining to the health care system. |
|  | *Nutrition Policy* | Guidelines and objectives pertaining to food supply and nutrition including recommendations for healthy diet. |
|  | *Government regulation* | Exercise of governmental authority to control conduct. |
|  | *Legislation* | Works consisting of the text of proposed or enacted legislation that may be in the form of bills, laws, statutes, ordinances, or government regulations. |
|  | *Legislation Food* | Laws and regulations concerned with industrial processing and marketing of foods and beverages. |
|  | *Voluntary programs* | Programs in which participation is not required. |
|  | *Mandatory programs* | Programs in which participation is required. |
|  | *Food labeling* | Use of written, printed, or graphic materials upon or accompanying a food or its container or wrapper. The concept includes ingredients, NUTRITIONAL VALUE, directions, warnings, and other relevant information. |
|  | *Product labeling* | Use of written, printed, or graphic materials upon or accompanying a product or its container or wrapper. It includes purpose, effect, description, directions, hazards, warnings, and other relevant information. |
|  | *Food Packaging* | Containers, packaging, and packaging materials for processed and raw foods and beverages. It includes packaging intended to be used for storage and also used for preparation of foods such as microwave food containers versus COOKING AND EATING UTENSILS. Packaging materials may be intended for food contact or designated non-contact, for example, shipping containers. FOOD LABELING is also available. |
|  | *Patient Protection and Affordable Care Act* | An Act prohibiting a health plan from establishing lifetime limits or annual limits on the dollar value of benefits for any participant or beneficiary after January 1, 2014. It provides that a health plan shall not be prevented from placing annual or lifetime per-beneficiary limits on covered benefits. |
| **Outcome** | *Food quality* | Ratings of the characteristics of food including flavor, appearance, nutritional content, and the amount of microbial and chemical contamination. |
|  | *Food* | Substances taken in by the body to provide nourishment. |
|  | *Beverages* | Liquids that are suitable for drinking. |
|  | *Food and Beverages* | Edible or potable substances. |
|  | *Food ingredients* | Substances included in prepared foods and beverages. |
|  | *Serving size* | A reference measure of food used to identify the calorie and nutrient content in a particular amount of that food. It is defined by an authoritative source, such as the Food Guide Pyramid devised by the UNITED STATES DEPARTMENT OF AGRICULTURE. |
|  | *Portion size* | The amount of a particular food one chooses to eat at a single meal. It is different from SERVING SIZE, which is a reference amount of food as defined by an authoritative source, such as the Food Guide Pyramid devised by the UNITED STATES DEPARTMENT OF AGRICULTURE. |
