# Supplementary material for: Effects of Menu Labeling Policies on Transnational Restaurant Chains to Promote a Healthy Diet: A Scoping Review to Inform Policy and Research
Source: Nutrients. 2020 May 26;12(6):1544. doi: 10.3390/nu12061544 (PMC7352298; doi:10.3390/nu12061544)
Supplement: Supplementary file 1 [file nutrients-12-01544-s001.zip › Supplementary material/Supplementary material 2.docx]

**Supplementary material 2.** Search details on each database

| **Database** | **Query** | **Items Found** |
| --- | --- | --- |
| PubMed | (((((("Restaurants"[MeSH] OR "Food Services"[MeSH] OR "Food Supply"[MeSH] OR "Fast Foods"[MeSH] OR "Food Industry"[MeSH] OR "Food-Processing Industry"[MeSH] “Chain restaurant*” OR restaurant or “food retail” OR “food services*” OR “food supply” OR “food supplies” OR “fast food*”)) NOT Schools [MeSH])) AND (((("Policy"[MeSH] OR "Nutrition Policy"[MeSH] OR "Public Policy"[MeSH] OR "Health Policy"[MeSH] OR "Government Regulation"[MeSH] OR "Legislation" [Publication Type] OR "Legislation, Food"[MeSH] OR "Voluntary Programs"[MeSH] OR "Mandatory Programs"[MeSH] OR "Patient Protection and Affordable Care Act"[MeSH] OR “Mandatory Policy” OR “Voluntary Policy” OR “Self-regulation” OR “Nutrition policies” OR Guideline OR “Food Policy”))) AND (("Food Labeling"[MeSH] OR "Product Labeling"[MeSH] OR “Food product label*” OR “Menu label*” OR “Restaurant label*” OR “Restaurant label” OR “Restaurant menu label*” OR “Food calories” OR “Nutrient label*” OR “Food content”) NOT “Food Packaging”))))) AND ((("Food"[MeSH] OR “Beverages”[MeSH] OR "Food and Beverages"[MeSH] OR "Food Ingredients"[MeSH] OR “food product*” OR “Fast food”)) AND ("Food Quality"[MeSH] OR "Food, Formulated"[MeSH] OR "Serving Size"[MeSH] OR "Portion Size"[MeSH] OR “Food reformulation” OR “Reduce* Portion*” OR “Reduce* size*” OR “Product reformulation”)) | 47 |
| CINHAL/EBSCO | ( ("Restaurants" OR "Food Services" OR "Food Supply" OR "Fast Foods" OR "Food Industry" OR "Food-Processing Industry" OR “Chain restaurant*” OR Restaurant or “Food Retail” OR “Food Services*” OR “Food Supply” OR “Food Supplies” OR “Fast food*” NOT Schools) ) AND TI ( ( "Policy" OR "Nutrition Policy" OR "Public Policy" OR "Health Policy" OR "Government Regulation" OR "Legislation" OR "Legislation, Food" OR "Voluntary Programs" OR "Mandatory Programs" OR "Patient Protection and Affordable Care Act" OR “Mandatory Policy” OR “Voluntary Policy” OR “Self-regulation” OR “Nutrition policies” OR Guideline OR “Food Policy” AND "Food Labeling" OR "Product Labeling" OR “Food product label*” OR “Menu label*” OR “Restaurant label*” OR “Restaurant label” OR “Restaurant menu label*” OR “Food calories” OR “Nutrient label*” OR “Food content” NOT “Food Packaging” ) ) AND TI ( ("Food" OR “Beverages” OR "Food and Beverages" OR "Food Ingredients" OR “food product*” OR “Fast food” AND "Food Quality" OR "Food, Formulated" OR "Serving Size" OR "Portion Size" OR “Food reformulation” OR “Reduce* Portion*” OR “Reduce* size*” OR “Product reformulation”) ) | 300 |
| Web of Science- | TS=(Restaurants OR “Food Services” OR “Food Supply” OR “Fast Foods” OR “Food Industry” OR “Food-Processing Industry” OR “Chain restaurant*” OR Restaurant or “Food Retail” OR “Food Services*” OR “Food Supply” OR “Food Supplies” OR “Fast food*” NOT Schools ) AND TS=(“Policy” OR “Nutrition Policy” OR “Public Policy” OR “Health Policy” OR “Government Regulation” OR “Legislation” OR “Legislation, Food” OR “Voluntary Programs” OR “Mandatory Programs” OR “Patient Protection and Affordable Care Act” OR “Mandatory Policy” OR “Voluntary Policy” OR “Self-regulation” OR “Nutrition policies” OR Guideline OR “Food Policy”) AND TS=(“Food Labeling” OR “Product Labeling” OR “Food product label*” OR “Menu label*” OR “Restaurant label*” OR “Restaurant label” OR “Restaurant menu label*” OR “Food calories” OR “Nutrient label*” OR “Food content” NOT “Food Packaging”) AND TS=(“Food” OR “Beverages” OR “Food and Beverages” OR “Food Ingredients” OR “food product*” OR “Fast food”) AND TS=(“Food Quality” OR “Food, Formulated” OR “Serving Size” OR “Portion Size” OR “Food reformulation” OR “Reduce* Portion*” OR “Reduce* size*” OR “Product reformulation”) | 113 |
| Google | Restaurants OR "Food Services" OR "Food Supply" OR "Fast Foods" OR "Food Industry" OR "Food-Processing Industry" OR “Chain restaurant*” OR Restaurant or “Food Retail” OR “Food Services*” OR “Food Supply” OR “Food Supplies” OR “Fast food*” NOT Schools AND "Policy" OR "Nutrition Policy" OR "Public Policy" OR "Health Policy" OR "Government Regulation" OR "Legislation" OR "Legislation, Food" OR "Voluntary Programs" OR "Mandatory Programs" OR "Patient Protection and Affordable Care Act" OR “Mandatory Policy” OR “Voluntary Policy” OR “Self-regulation” OR “Nutrition policies” OR Guideline OR “Food Policy” AND "Food Labeling" OR "Product Labeling" OR “Food product label*” OR “Menu label*” OR “Restaurant label*” OR “Restaurant label” OR “Restaurant menu label*” OR “Food calories” OR “Nutrient label*” OR “Food content” NOT “Food Packaging” AND "Food" OR “Beverages” OR "Food and Beverages" OR "Food Ingredients" OR “food product*” OR “Fast food” AND "Food Quality" OR "Food, Formulated" OR "Serving Size" OR "Portion Size" OR “Food reformulation” OR “Reduce* Portion*” OR “Reduce* size*” OR “Product reformulation” | 6,340 results  Only first 100 hints were sorted by relevance |

*PubMed* (with MEDLINE) is a premiere biomedical research database and is a service of the National Library of Medicine that includes over 19 million citations. *CINAHL* is a cumulative Index to Nursing and Allied Health Literature – citations, abstracts, and full text from journals in nursing and allied health from 1981 to present. *Web of Science* is a database index citation from journal articles and conferences in the social sciences, arts, and humanities. *Google Scholar* is a search engine for scholarly research that combine the quality of sources from diverse library databases.
