# Supplementary material for: Effects of Menu Labeling Policies on Transnational Restaurant Chains to Promote a Healthy Diet: A Scoping Review to Inform Policy and Research
Source: Nutrients. 2020 May 26;12(6):1544. doi: 10.3390/nu12061544 (PMC7352298; doi:10.3390/nu12061544)
Supplement: Supplementary file 1 [file nutrients-12-01544-s001.zip › Supplementary material/Supplementary material 3.docx]

| Year, Author | 1. Were the criteria for inclusion in the sample clearly defined? | | 2. Were the study subjects/objects and the settings described in detail? | | 3. Was menu labeling measured in a valid reliable way? | | 4. Were objective, standard criteria used for measurement of menu labeling? | | 5. Were cofounders and/or covariates identified? | | 6. Were strategies to deal, adjust cofounders and/or covariates stated? | | 7. Were food reformulation, nutrient composition, serving size portion reduction measured in a valid and reliable way? | | 8. Was appropriate statistical analysis used? | | Overall appraisal score | |
| --- | --- | --- | --- | --- | --- | --- | --- | --- | --- | --- | --- | --- | --- | --- | --- | --- | --- | --- |
| Reviewers | **R1** | **R2** | **R1** | **R2** | **R1** | **R2** | **R1** | **R2** | **R1** | **R2** | **R1** | **R2** | **R1** | **R2** | **R1** | **R2** | **R1** | **R2** |
| Bleich et al. 2015 | 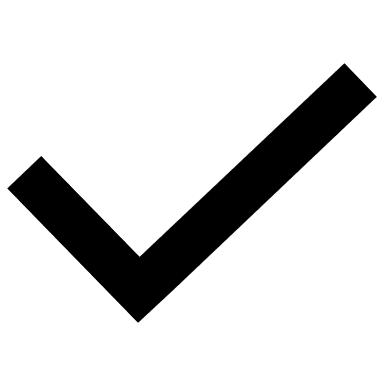 | 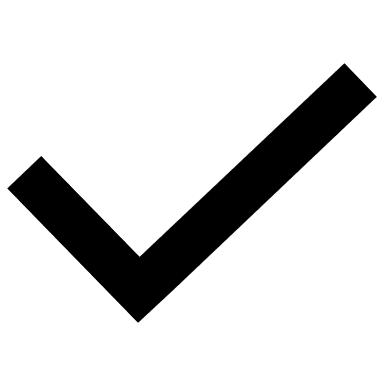 | 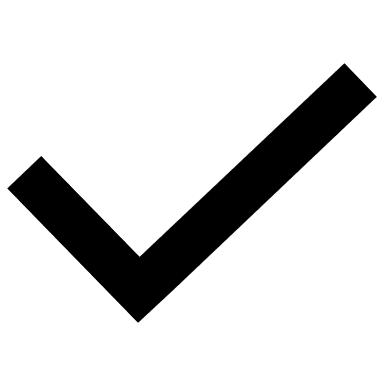 | 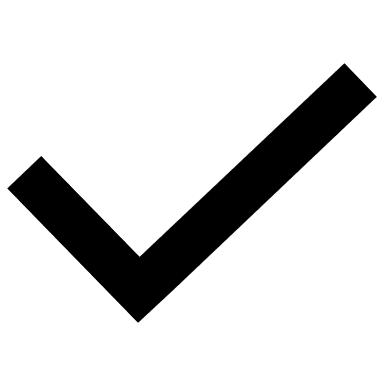 | 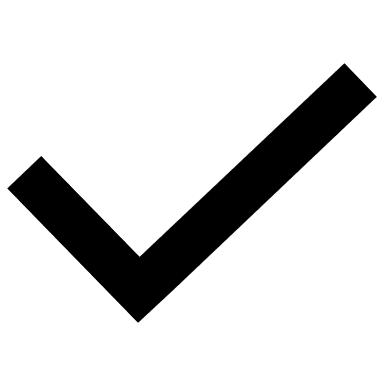 | 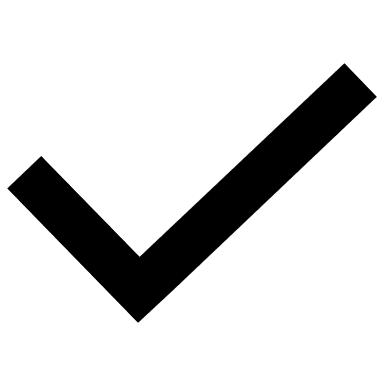 | 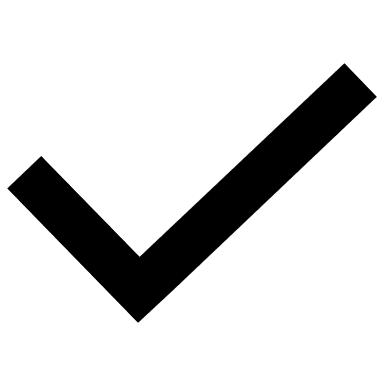 | 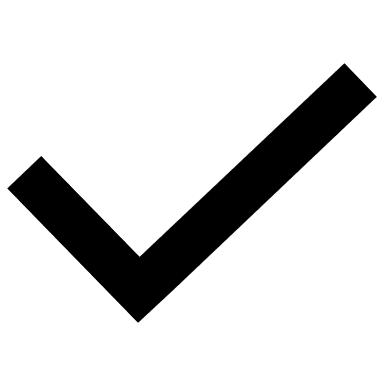 | 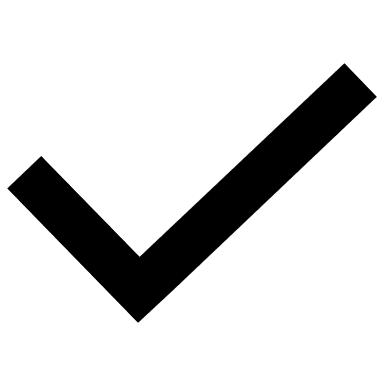 | 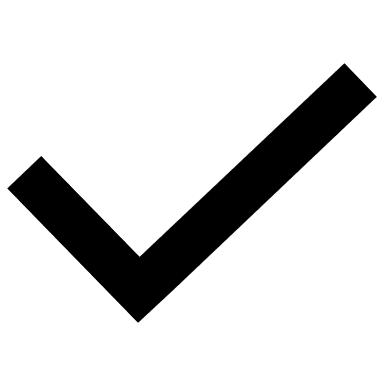 | 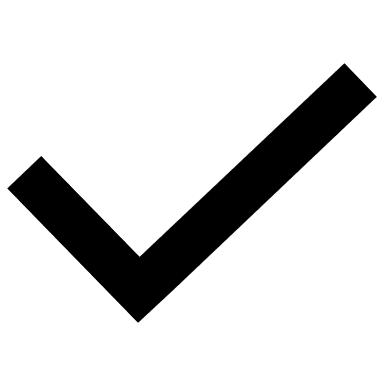 | 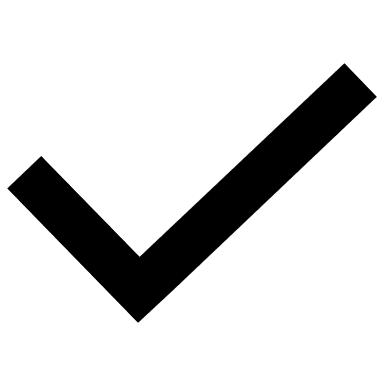 | 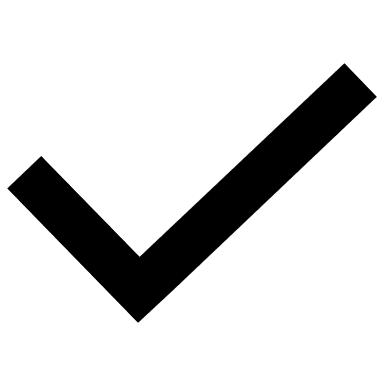 | 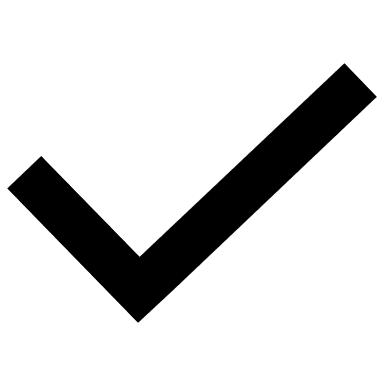 | 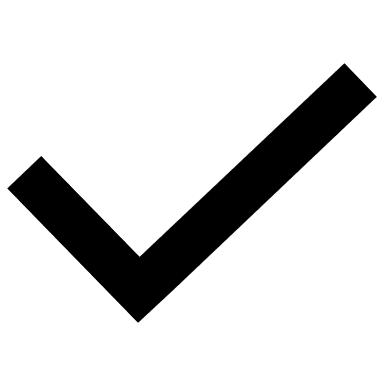 | 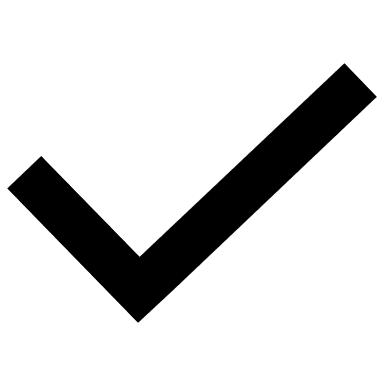 | Good | Good |
| Bleich et al. 2016 | 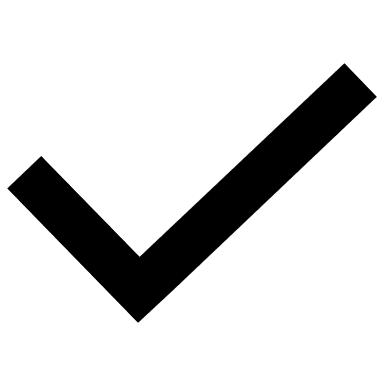 | 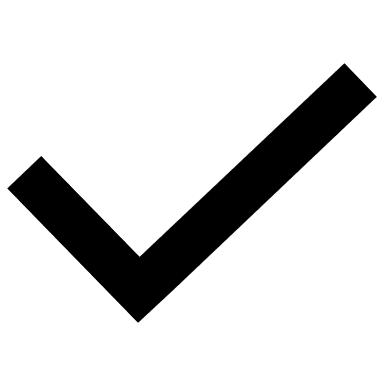 | 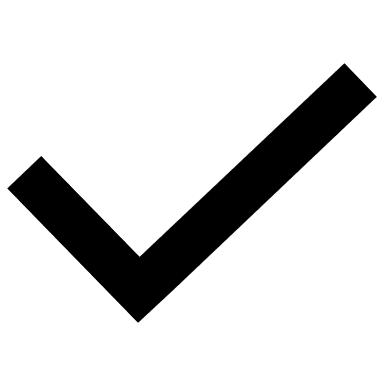 | 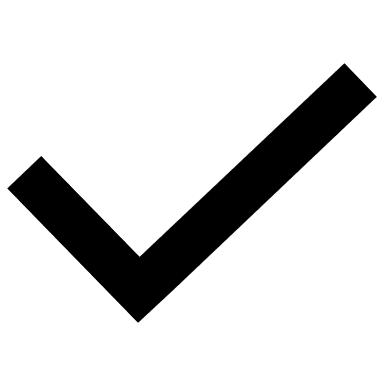 | 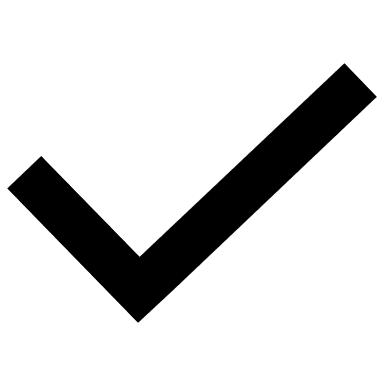 | 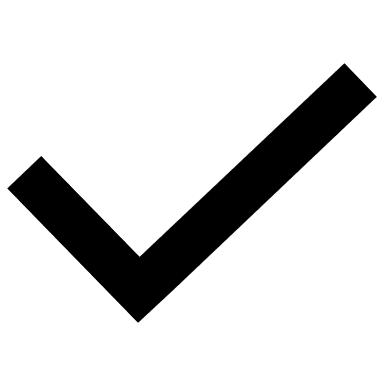 | 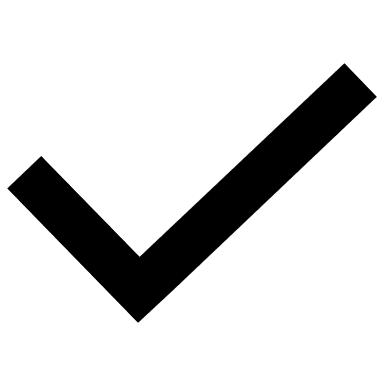 | 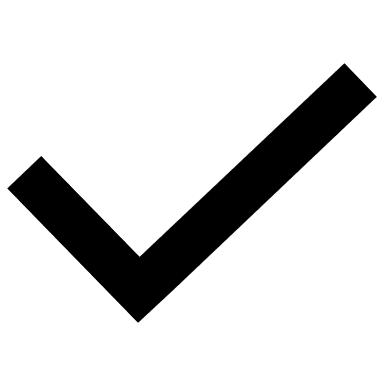 | 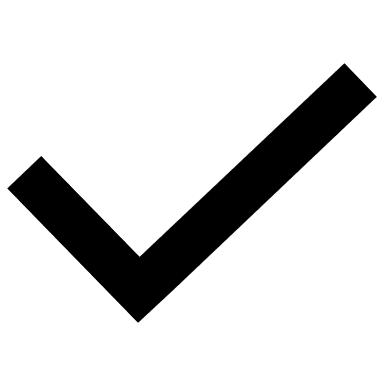 | 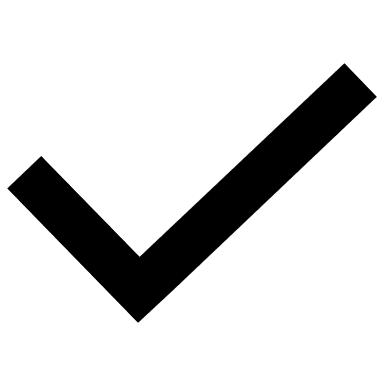 | 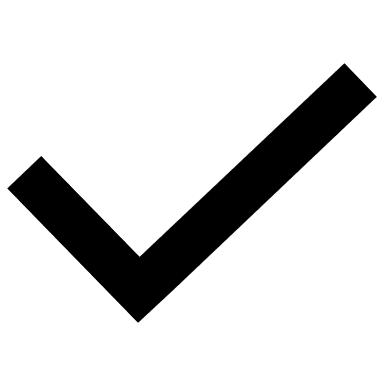 | 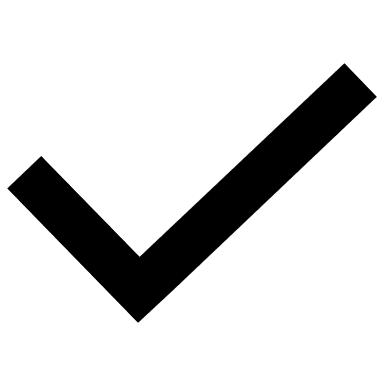 | 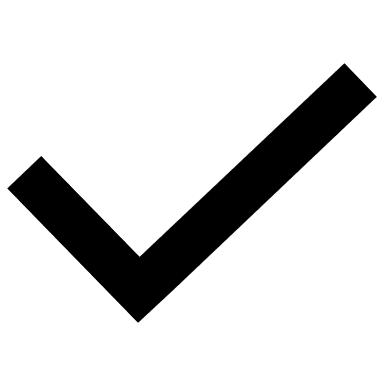 | 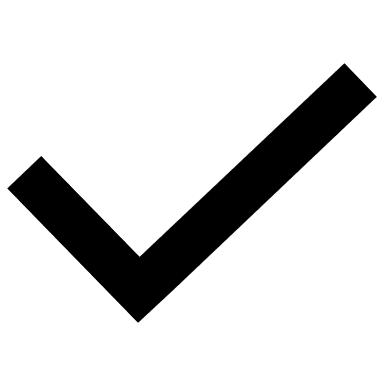 | 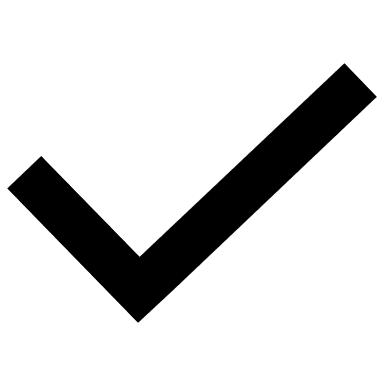 | 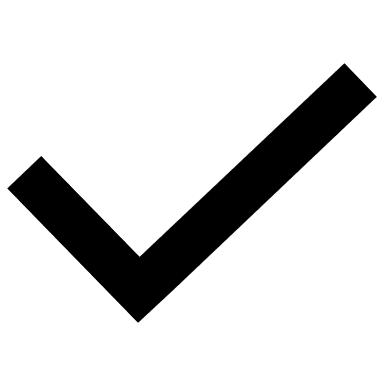 | Good | Good |
| Bleich et al. 2017 | 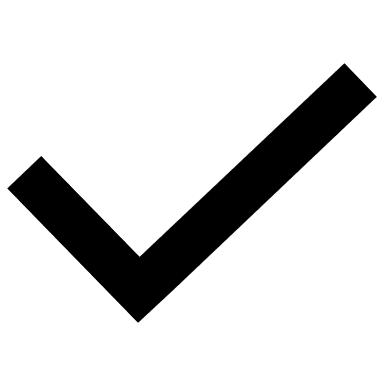 | 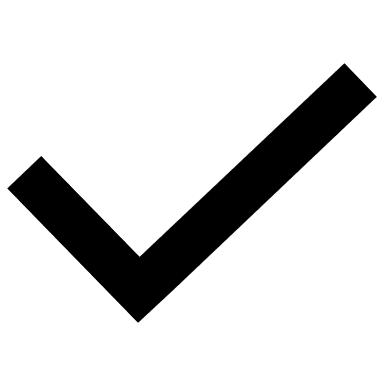 | 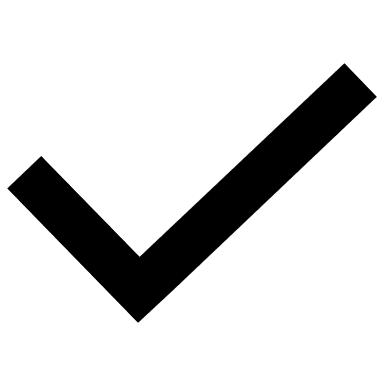 | 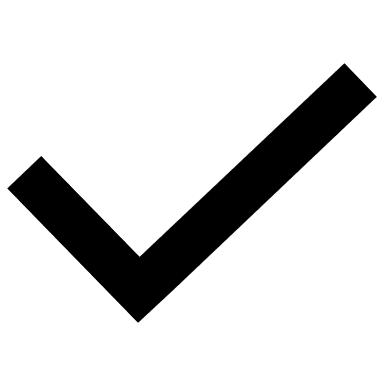 | 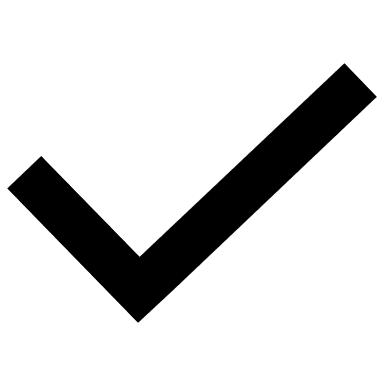 | 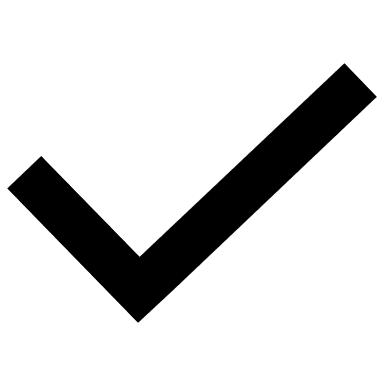 | 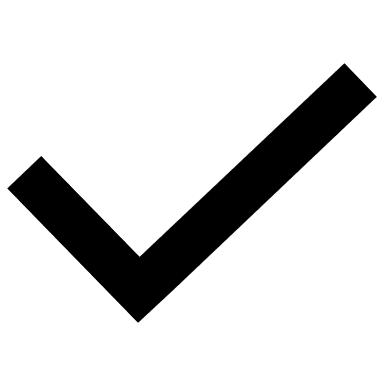 | 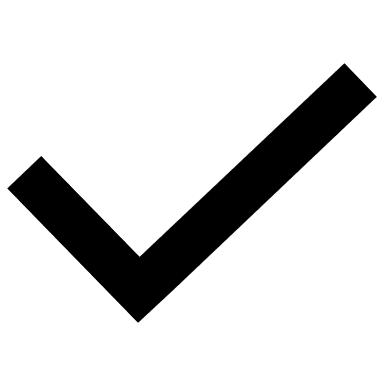 | 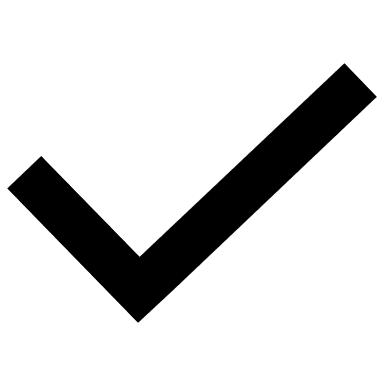 | 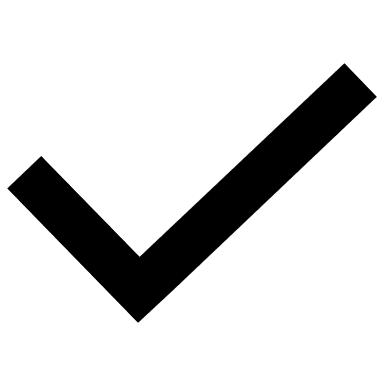 | 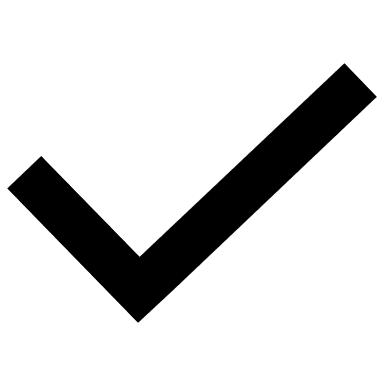 | 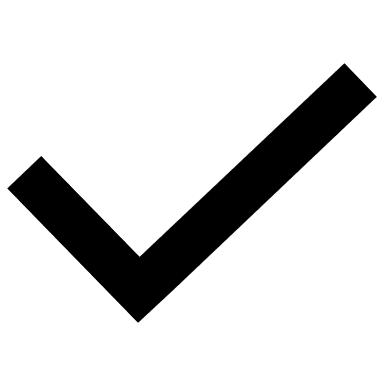 | 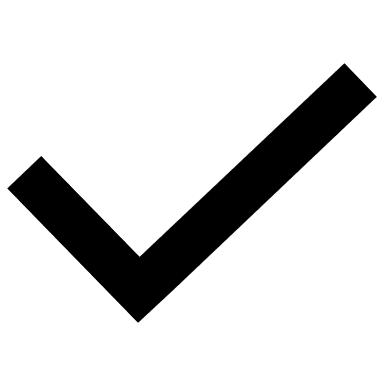 | 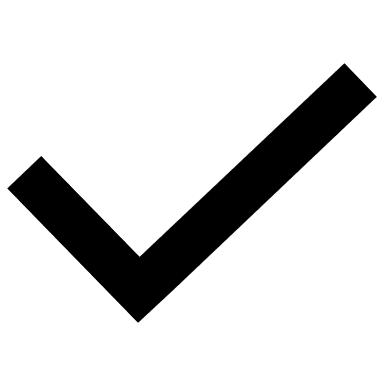 | 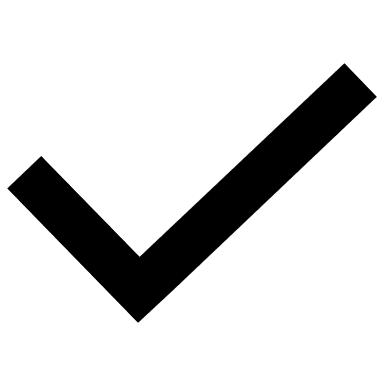 | 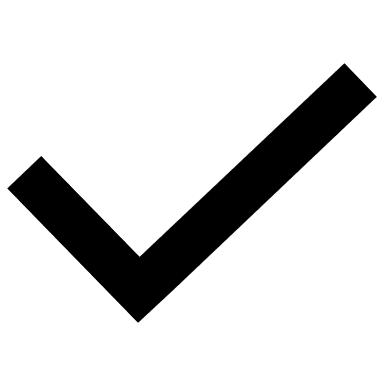 | Good | Good |
| Bleich et al. 2018 | 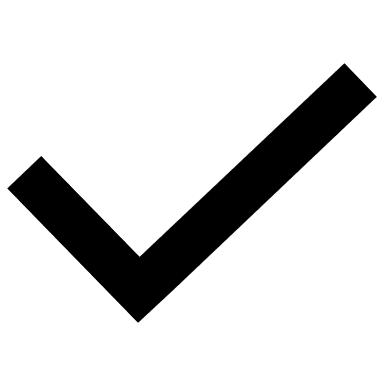 | 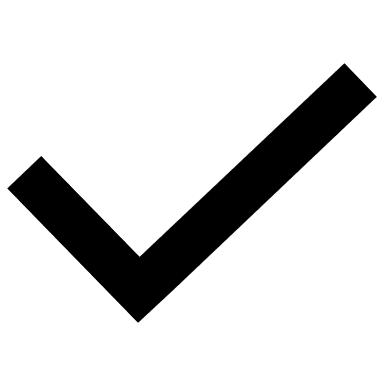 | 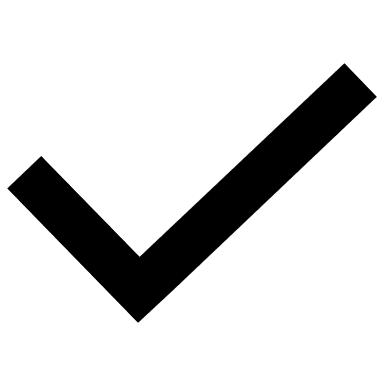 | 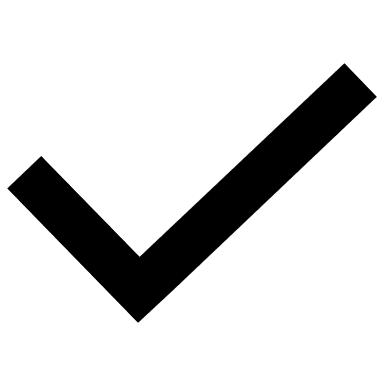 | 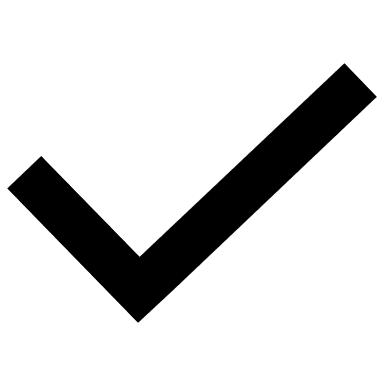 | 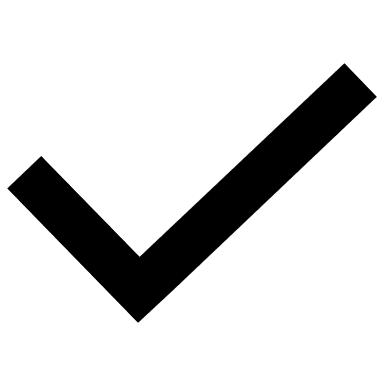 | 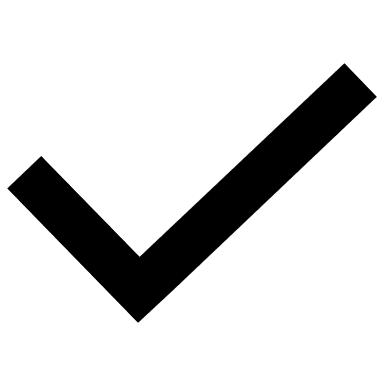 | 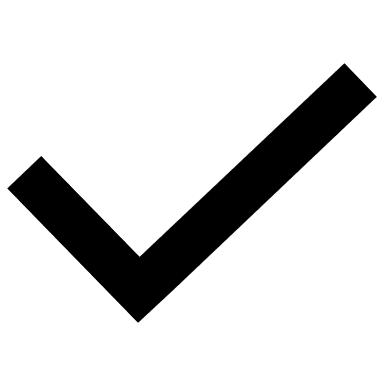 | 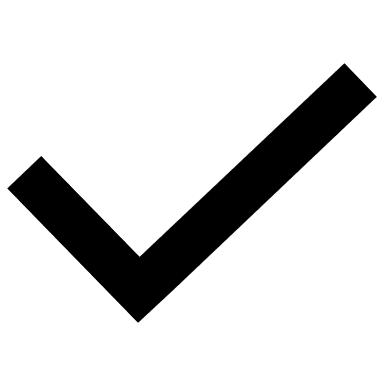 | 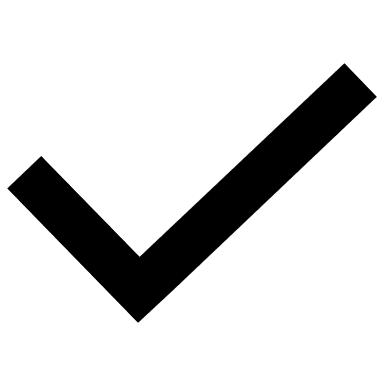 | 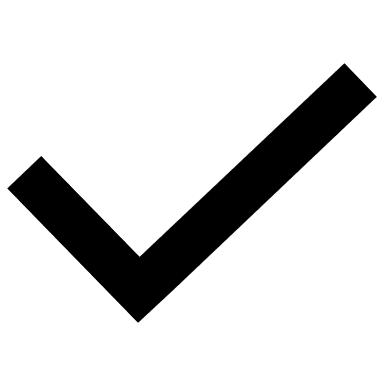 | 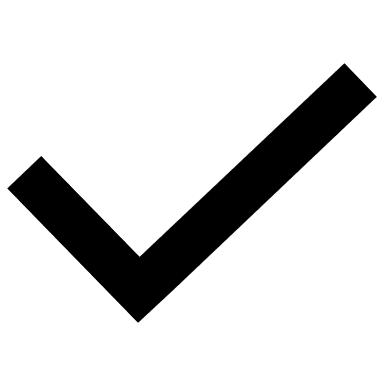 | 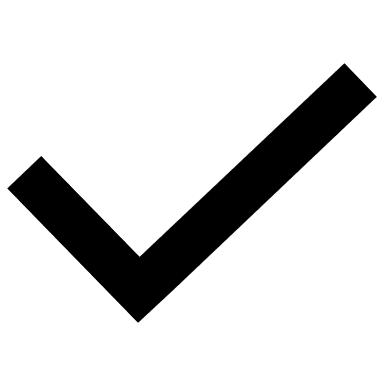 | 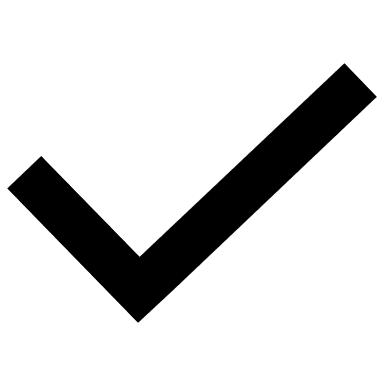 | 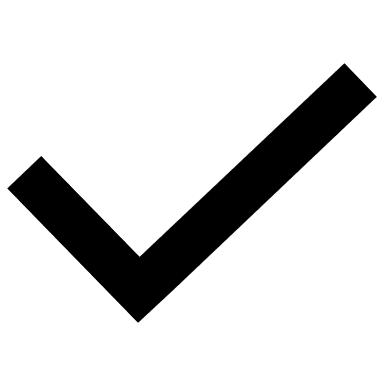 | 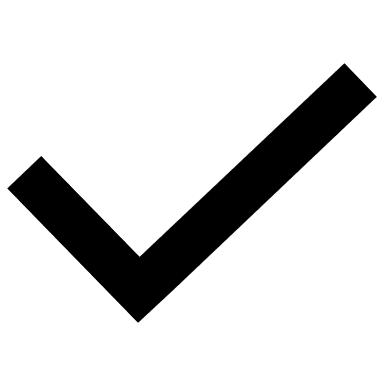 | Good | Good |
| Bleich et al. 2020 | 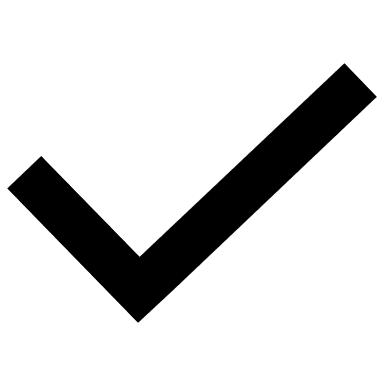 | 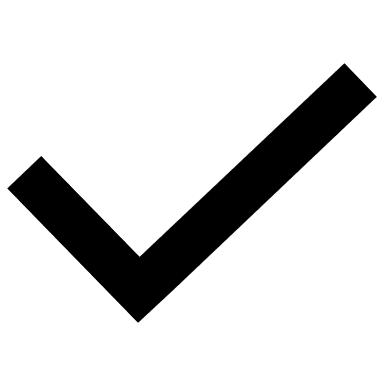 | 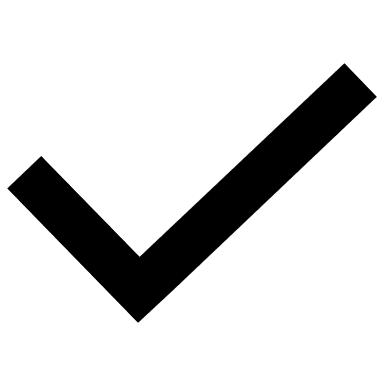 | 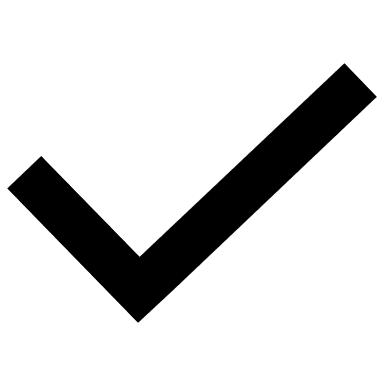 | 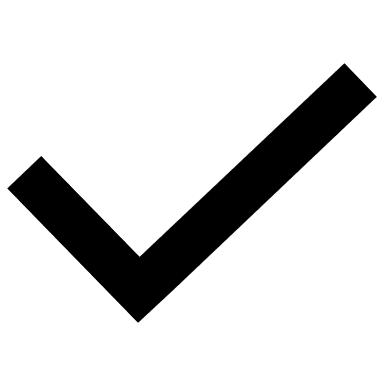 | 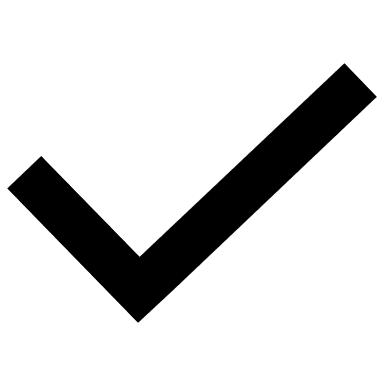 | 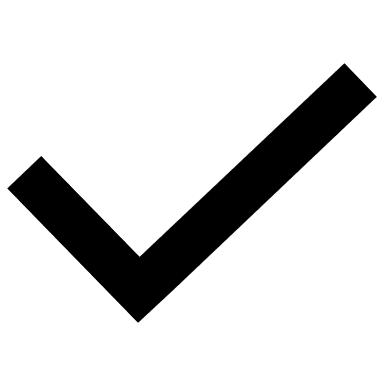 | 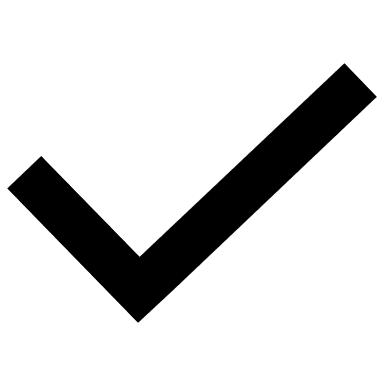 | 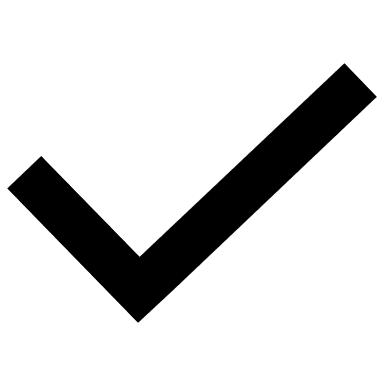 | 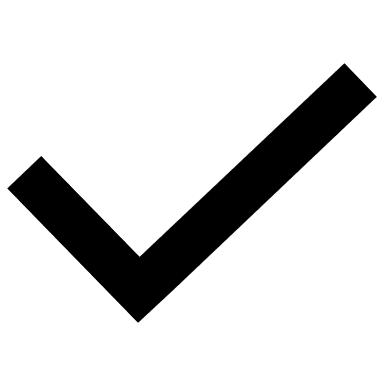 | 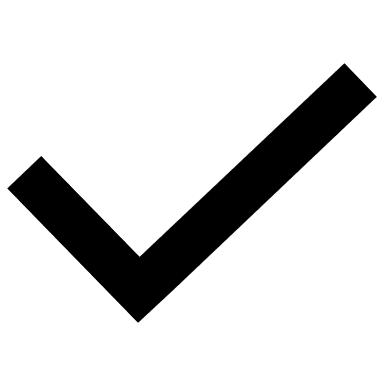 | 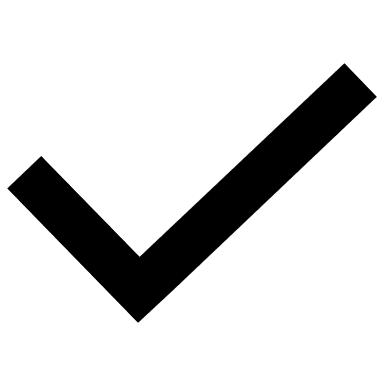 | 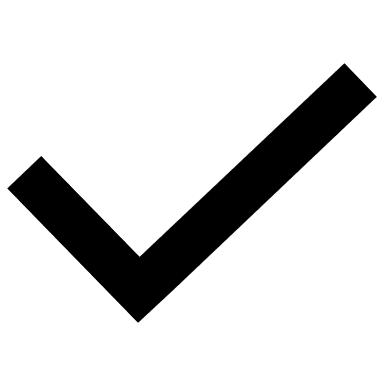 | 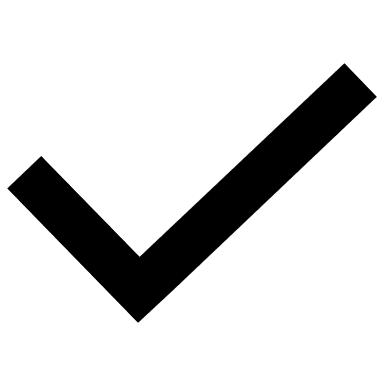 | 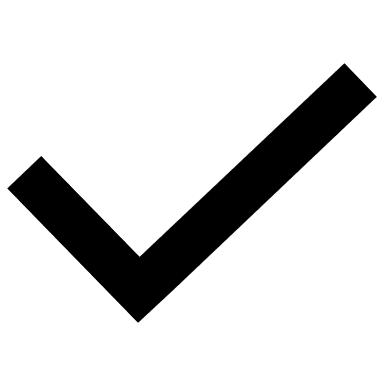 | 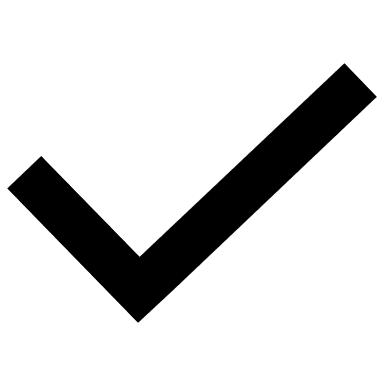 | Good | Good |
| Bruemmer et al. 2012 | 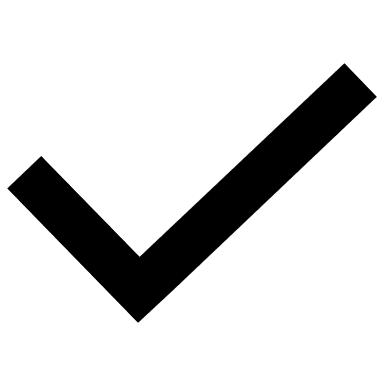 | 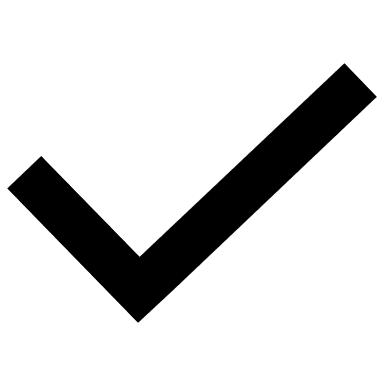 | 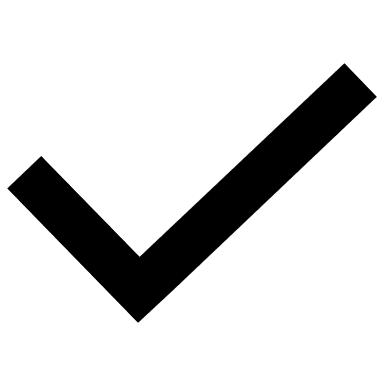 | 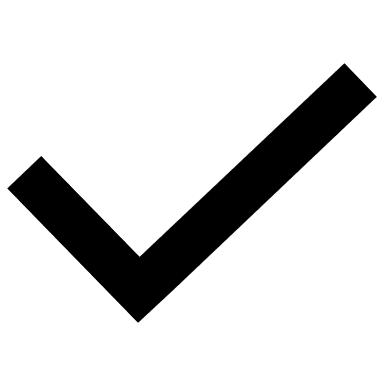 | 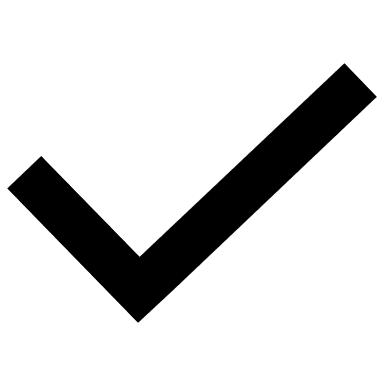 | 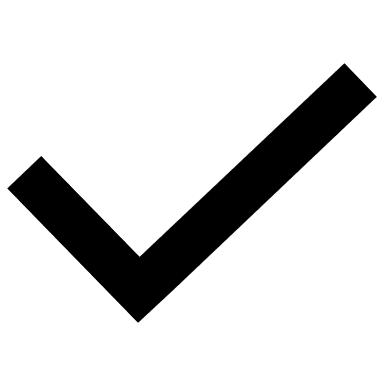 | 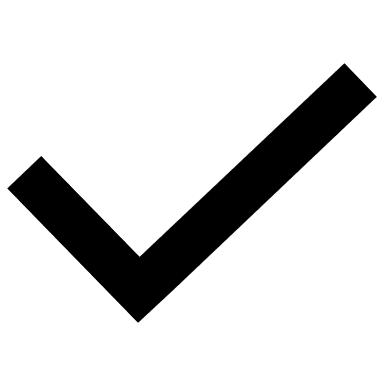 | 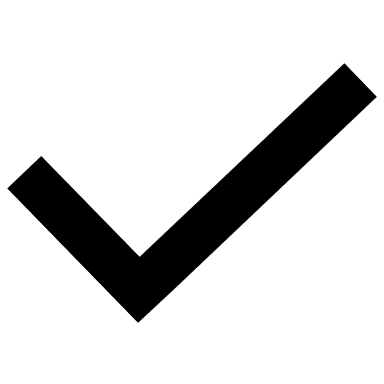 | **U** | **U** | **U** | 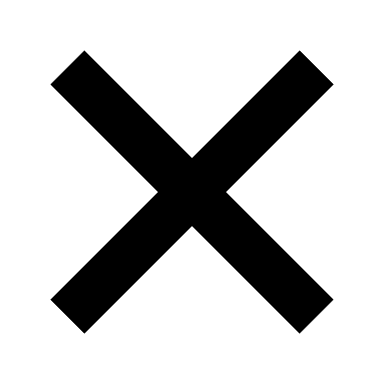 | 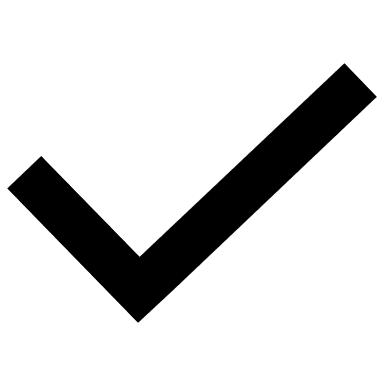 | 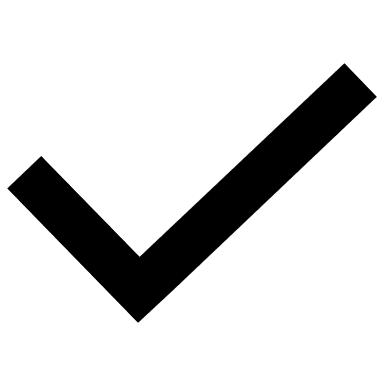 | 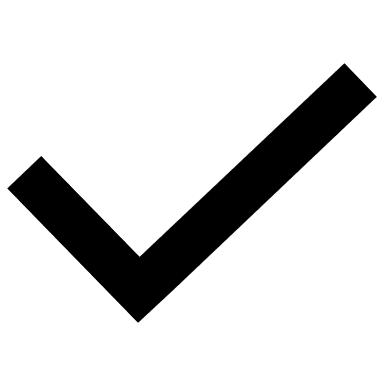 | 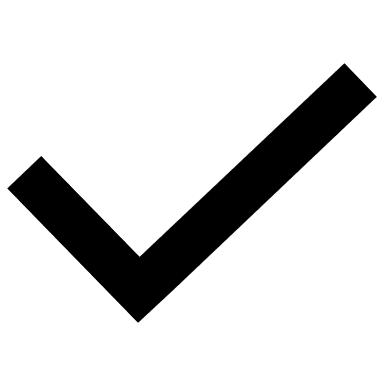 | Fair | Fair |
| Namba et al. 2013 | 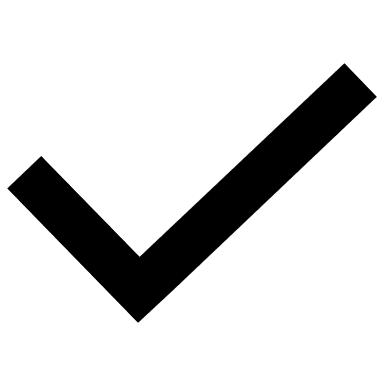 | 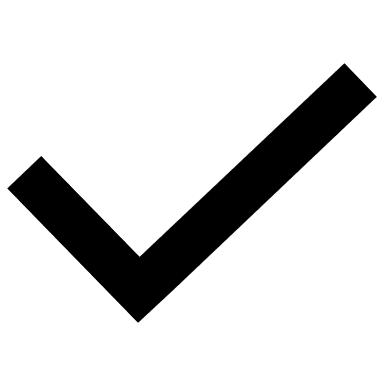 | 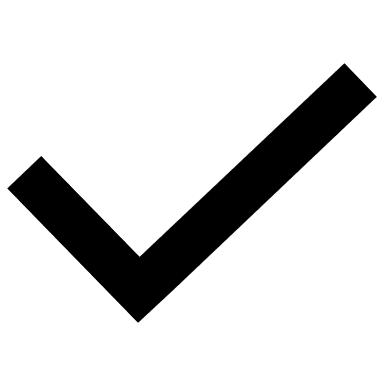 | 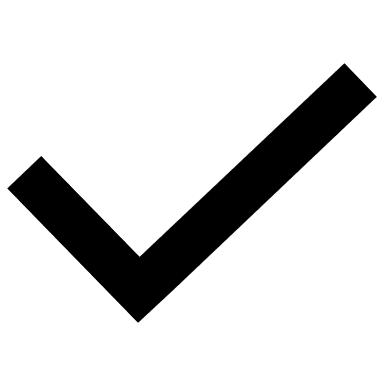 | 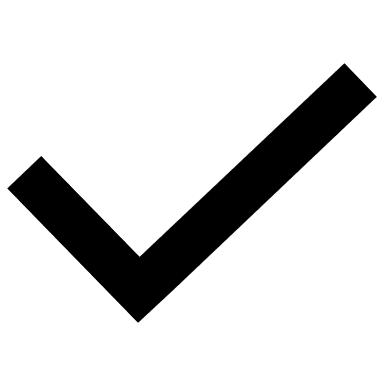 | 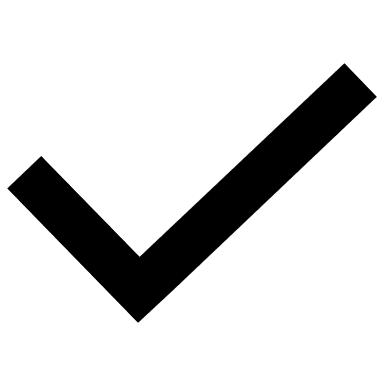 | 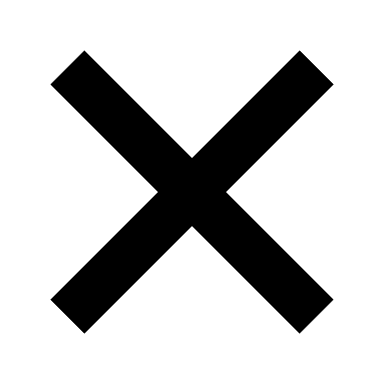 |  |  |  |  |  |  |  |  |  | Fair | Good |
| Petimar et al. 2019 |  |  |  |  |  |  |  |  |  |  |  |  |  |  |  |  | Good | Good |
| Saelens et al. 2012 | **U** |  |  |  |  |  |  |  | **U** | **U** | **U** |  |  | **U** |  |  | Fair | Fair |
| Scourboutakos et al. 2019 |  |  |  |  |  |  |  |  |  |  |  |  |  |  |  |  | Good | Good |
| Theis et al. 2019 |  |  |  |  |  |  |  |  |  |  |  |  |  |  |  |  | Good | Good |
| Tran et al. 2019 |  |  |  |  |  |  |  |  |  |  |  |  |  |  |  |  | Good | Good |
| Wellard-Cole et al. 2018 |  |  |  |  |  |  |  |  |  |  |  |  |  |  |  |  | Good | Good |
| Wellard-Cole et al. 2019 |  |  |  |  |  |  |  |  | **U** | **U** | **U** |  |  |  |  |  | Fair | Fair |
| Wu et al. 2014 |  |  |  |  |  |  |  |  |  |  |  |  |  |  |  |  | Good | Good |

**Supplementary material 3.** Quality assessment results based on the Johanna Briggs Institute critical appraisal checklist

The Johana Briggs Institute. *Checklist for Analytical Obesravtional Studies*; Australia, 2017

Overall appraisal score: “Poor” if more than 4 (50%) answers are *no*; “Fair” if less than 4 but more than 1 answers are *no* or *unclear*; “Good” if no answers are *no* or *unclear*. = yes; = no; **U**= unclear.
